# Spatial and temporal genetic homogeneity of the Monterey Spanish mackerel, *Scomberomorus concolor*, in the Gulf of California

**DOI:** 10.7717/peerj.2583

**Published:** 2016-10-25

**Authors:** Erika Magallón-Gayón, Pindaro Diaz-Jaimes, Manuel Uribe-Alcocer

**Affiliations:** 1 Posgrado en Ciencias del Mar y Limnología, Instituto de Ciencias del Mar y Limnología, Universidad Nacional Autónoma de México, Ciudad de México, Mexico; 2 Unidad Académica de Ecología y Biodiversidad Acuática, Instituto de Ciencias del Mar y Limnología, Universidad Nacional Autónoma de México, Ciudad de México, Mexico

**Keywords:** Population genetics, Historical demography, Microsatellites, *mtDNA*, Fisheries, Gene flow

## Abstract

The genetic homogeneity of the Monterey Spanish mackerel *Scomberomorus concolor* population in the Gulf of California was confirmed using nine nuclear microsatellite loci in combination with mitochondrial cytochrome b gene sequences. Samples were collected from the upper and central Gulf areas, representing the two main biogeographical regions of the Gulf. The analyses support the existence of a single panmictic population of *S. concolor* inhabiting the Gulf of California which in terms of fishery management represents a single genetic stock. Additionally, the contemporary effective population size estimated for the *S. concolor* population (Ne = 3056.9) was high and similar to another pelagic species. The gene flow seems to be bidirectional between the upper and central Gulf, which coincides with the seasonal movements between both regions related to spawning and feeding activities. A population expansion event was detected, which agrees with a colonization-expansion hypothesis of the *S. concolor* population in the Gulf.

## Introduction

The Gulf of California (GC) is a long, narrow, semi-isolated basin which was formed by tectonic rifting, separating the peninsula of Baja California from the mainland approximately 5–12 million years ago (Carreño & Helenes, 2002). Based on the environment and changes in the ichthyofaunal composition, the GC is divided into two distinct biogeographic regions, the upper and the central Gulf (Riginos, 2005). The upper Gulf is shallow, has extreme tidal heights (up to 10 m) and a wide range of sea surface temperatures (SST) (e.g., low temperature in winter and high temperature in summer) (Brusca et al., 2005). In contrast, the central part is deeper (with oceanic channels and canyons) with tide and temperature variations less pronounced than the upper Gulf (Thomson, Findley & Kerstich, 2000). A Midriff Archipelago Region (MAR) containing several islands of different sizes, islets, straits, basins and sills, has been proposed to affect the exchange of sea water between regions (Danell-Jiménez et al., 2009; Sánchez-Velasco et al., 2009) limiting the dispersal of individuals (Riginos, 2005). This has resulted in significant genetic differences between populations of both biogeographic regions for some reef fish species (Riginos & Nachman, 2001; Riginos & Victor, 2001).

Although the Monterey Spanish mackerel *Scomberomorus concolor* (Lockington, 1879) is an epipelagic fish species inhabiting these two main biogeographical regions of the Gulf of California (Collette & Nauen, 1983), the contrasting oceanographic conditions of the upper and central Gulf and the ocean circulation may play a main role in the habitat use and dispersal capability at different stages (larval or adult). The knowledge of the extent of gene flow between both regions is important to define the existence or not, of a panmictic population into the Gulf.

Previous mitochondrial DNA (*mtDNA*) sequence data from Domínguez-López et al. (2015) reported a single panmictic population of *S. concolor*, suggesting the existence of a mobile population migrating between the upper and central Gulf. However, the *mtDNA* is sensitive to past fluctuations in population size, which may obscure the signal of divergence (Eytan & Hellberg, 2010). Furthermore, the use of highly variable regions as the mitochondrial control region (*mtDNA-CR*) may result in complex and unclear phylogeographic patterns. As evidence of population expansion events and a high genetic variability for *S. concolor* has been previously reported, the use of a less variable region as the mitochondrial cytochrome b gene (*mtCyt-b*) may be useful to clarify the species’ phylogeography. This, besides the inclusion of nuclear microsatellites markers may offer a more complete scenario as it gathers information from both, maternal and paternal lineages within a more contemporary context. This approach might be useful to test the genetic homogeneity hypothesis of *S. concolor* population or alternatively, whether its migratory capability is insufficient to cross the MAR separating the upper and central Gulf.

*Scomberomorus concolor* is thought to have had a continuous distribution from Monterey Bay, California along the peninsula of Baja California up to the Gulf (Collette et al., 2011). However, the species is believed to have disappeared from the California coast and currently, *S. concolor* is present only in the upper and the central Gulf (Riginos, 2005). Because of this assumed reduction in its range, *S. concolor* has been included in the IUCN red list as a vulnerable species (Collette et al., 2011). *S. concolor* supports a well-established fishery in the GC together with its sister species *Scomberomorus sierra*. Some studies have stated that the species is at risk of collapse (Quiñónez-Velázquez & Montemayor-López, 2002) or near to its maximum level of exploitation (Valdovinos-Jacobo, 2006). However, no fishery regulation nor conservation measures for this species exist. Thereby, one relevant issue for the management of the fishery is whether the *S. concolor* consists of a single genetic stock distributed in both Gulf regions. For this purpose, we used nuclear DNA in addition to the *mtCyt-b*, to assess the population genetic structure of *S. concolor*, to evaluate current levels of gene flow in the Gulf and to elucidate the species’ phylogeography.

## Materials and Methods

### Biological materials

**Figure 1:**
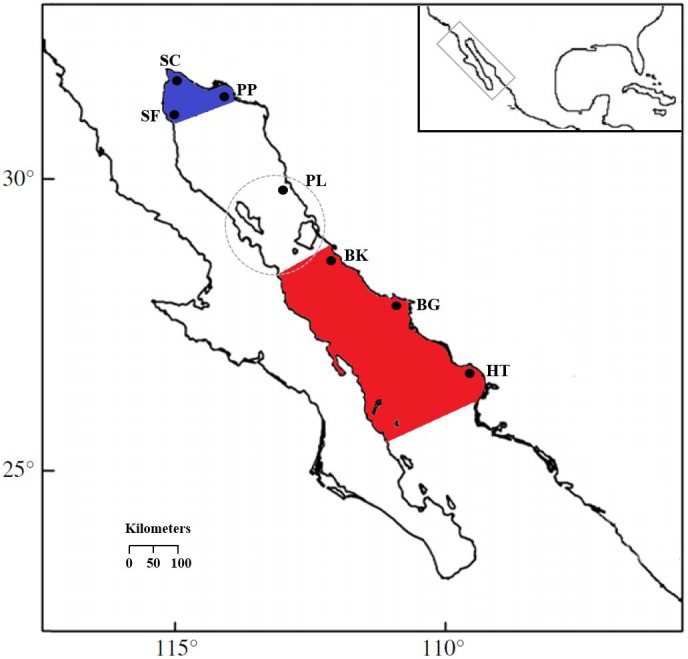
Locations of *S. concolor* samples in the Gulf of California. Upper Gulf: San Felipe (SF), Santa Clara (SC), Puerto Peñasco (PP) and Puerto Libertad (PL). Central Gulf: Bahía Kino (BK), Bahía Guaymas (BG) and Huatabampo (HT). Upper Gulf colored in blue, central Gulf colored in red, the dashed circle surrounds the Midriff Archipelago Region (MAR).

Muscle tissue samples were collected from *S. concolor* individuals caught by artisanal fishing boats, for that reason no collection permits were needed. There were 482 Mackerels obtained at the two major distribution areas in the GC over a period of four years (Table 1 and see also Table S1) and tissue samples were stored in 70% ethyl alcohol. The sampling locations in the upper Gulf included San Felipe in 2006 (SF06; *n* = 37) and 2008 (SF08; *n* = 32), Santa Clara in 2006 (SC; *n* = 29), Puerto Peñasco in 2006 (PP06; *n* = 29), 2007 (PP07; *n* = 15) and 2008 (PP08; *n* = 32), and Puerto Libertad in 2006 (PL; *n* = 30). The central Gulf locations were Bahía Kino in 2005 (BK05; *n* = 50), in 2006 (BK06; *n* = 60), and 2007 (BK07; *n* = 31), Bahía Guaymas in 2005 (BG05; *n* = 70), and 2006 (BG06; *n* = 39) and Huatabampo in 2005 (HT; *n* = 28) (Fig. 1). The samples were obtained during the fishing season at the upper and central regions, respectively (Table 1). It is important to remark that due to the migratory nature of *S. concolor*, temporal collections were included as well as simultaneous sampling for both GC areas in order to test the genetic homogeneity hypothesis for a mobile population. Even though more sampling sites were visited, in many of these fishing sites the catches are sporadic or incidental.

**Table 1:** Sampling of *S. concolor* individuals collected from artisanal fisheries in the Gulf of California. The total sample size was of 482 individuals. The *mtCyt-b* sequences were obtained only for the location codes marked in bold.

Region	Location	Date of collection	Location code	Sample size	Latitude	Longitude
Upper Gulf (UG)	San Felipe	2006^*^	**SF06**	37	31°01′39N	114°50′07W
		May 2008	SF08	32		
	Santa Clara	April 2006	**SC06**	29	31°41′12N	114°29′59W
	Puerto Peñasco	September 2006	**PP06**	29	31°19′00N	113°32′13W
		August 2007	PP07	15		
		April 2008	PP08	32		
	Puerto Libertad	April 2006	**PL06**	30	29°54′15N	112°40′59W
Central Gulf (CG)	Bahía Kino	April 2005	BK05	50	28°49′22N	111°56′27W
		April 2006	**BK06**	60		
		February 2007	BK07	31		
	Bahía Guaymas	March 2005	**BG05**	70	27°55′06N	110°53′56W
		April 2006	BG06	39		
	Huatabampo	April 2005	**HT05**	28	26°49′39N	119°38′32W

**Notes.**

* Month not available.

### DNA analysis

Total genomic DNA for each sample was isolated using the proteinase K-lysis buffer extraction protocol (Laird et al., 1991). Nine microsatellite loci were amplified by polymerase chain reaction (PCR) using primers previously developed for *Scomberomorus cavalla* (Broughton, Stewart & Gold, 2002), *Scomberomorus brasiliensis* (Renshaw et al., 2009) and *Scomberomorus niphonius* (Yokoyama et al., 2006). We used a three-primer strategy for each PCR reaction consisting of forward and reverse primers, the former containing a M13-tail (5′-GTAAAACGA CGGCCAGT-3′) at its 5′ end, plus a fluorescently labelled universal M13 primer (Schuelke, 2000). PCR amplifications used the Type-it Microsatellite PCR Kit (QIAGEN™; Hilden, Germany; Cat. No. 206243) in 5 µL reactions containing 10–50 ng of DNA, primer mix (forward and reverse primers at 0.2 µM and universal M13 primer at 0.4 µM in RNase-free water), and Type-it Master Mix (0.4X) (containing HotStarTaq^®^ *Plus* DNA Polymerase, Type-it Microsatellite PCR Buffer with 3 mM MgCl_2_, and dNTPs) and RNase-free water. The high specificity of the kit components allowed multiplex PCR reactions. For this reason, loci were organized into four multiplex sets per PCR reaction that permitted to separate loci with overlapping allelic size ranges (Table S2). Cycling conditions consisted of an initial denaturation step at 95 °C for 5 min followed by 28 cycles of denaturation at 95 °C for 30 sec, annealing at 56 °C for 90 sec, extension at 72 °C for 30 sec, and a final extension at 60 °C for 30 min. PCR products were electrophoresed in a capillary sequencer ABI 3500 (Applied Biosystem, Carlsbad, California, USA) using a GeneScan 500-LIZ size standard (Applied Biosystem). Fragment lengths were assessed with GeneMapper^®^ 4.1 software (Life Technologies). Genotype determination was based on the allelic size range for each locus (Broughton, Stewart & Gold, 2002; Yokoyama et al., 2006; Renshaw et al., 2009).

The entire *mtCyt-b* gene of *S. concolor* was amplified in ten samples with the primers Gludg-L (5′-TGACTTGAARAACCAYCGTTG-3′) (Palumbi et al., 2002) and revThrRF (5′-TCCGACATCTGGATTACAA-3′) (Rocha-Olivares, Rosenblatt & Vetter, 1999) and sequenced with the primer Gludg-L. These sequences were used in order to design an internal primer (CytB-I: 5′-CGCCGATTCAGGTAAGGATA-3′), which was subsequently used to amplify and sequence along with the Gludg-L primer a fragment of 873 base pairs (bp) for the rest of samples. Additionally, thirteen *mtCyt-b* sequences of *S. sierra* were sequenced for further analysis (Genbank accession numbers: KX462440– KX462527). PCR reactions were performed with DreamTaq DNA Polymerase (Cat. No. EP0701; Thermo Scientific, Sao Paulo, Brazil) in 15 µL reactions containing 10–100 ng of DNA, DreamTaq Buffer (10X) (containing KCl, (NH_4_)_2_SO_4_ and 20 mM MgCl_2_), 0.2 mM dNTPs, 0.2 µM of each primer, 0.38 U of DreamTaq^®^ DNA polymerase and deionized water. Cycling conditions consisted of an initial denaturation step at 95 °C for 5 min, followed by 35 cycles of denaturation at 95 °C for 1 min, annealing at 56 °C for 1 min and an extension at 65 °C for 3 min, and a final extension at 72 °C for 7 min. The resulting PCR products were purified and sequenced in an ABI 3730xl (Applied Biosystem) automatic sequencer using primer CytB-I.

### Data analyses

The software Micro-Checker 2.2 (Van Oosterhout et al., 2004) was used to identify possible genotyping errors (i.e., null alleles, stuttering and large allele dropout) within the microsatellite data.

The number of alleles and allelic richness per locus were calculated in FSTAT 2.9 (Goudet, 1995), based on a weighted sample of 24 individuals. Deviations from Hardy-Weinberg equilibrium and estimates of observed (*H*_*o*_) and expected (*H*_*E*_) heterozygosity were estimated with Arlequin 3.5 (Excoffier & Lischer, 2010). Heterozygosity excess or deficit and single locus *F*_*IS*_ estimates, were calculated using Genepop 4.2 software (Raymond & Rousset, 1995; Rousset, 2008). Arlequin was used to estimate the linkage disequilibrium probability values for the observed allelic association under the null hypothesis of random allelic assortment.

As *F*_*ST*_ and *G*_*ST*_ can underestimate genetic structure when using highly polymorphic markers such as microsatellites, an analysis using CoDiDi software (Wang, 2015) was performed to assess whether the microsatellite loci were appropriate for *F*_*ST*_ comparisons. This program calculates the correlation coefficient (*r*_*GH*_) between *G*_*ST*_ and *H*_*S*_ (expected heterozygosity) across all loci and test its significance (Wang, 2015). Afterwards, to test significance of interannual variation in allele frequencies, we performed *F*_*ST*_ pairwise sample comparisons over all temporal collections. After testing for temporal genetic differences between collections, samples were pooled into San Felipe (SF), Puerto Peñasco (PP), Bahía Kino (BK) and Bahía Guaymas (BG), in order to increase the statistical power for testing significance in spatial variation through *F*_*ST*_ pairwise sample comparisons.

A structure analysis was performed using STRUCTURE 2.3.4 (Pritchard, Stephens & Donnelly, 2000). We conducted three replicate runs for *k* = 1 to 7, where *k* = 1 means no population differentiation and *k* = 7 corresponds to the maximum number of sampled populations. Each run consisted of a 500,000 MCMC after a burn-in period of 50,000. We tested with and without admixture model, both with the correlated allele frequency model. The posterior probability LnP(D) was used to infer the most probable number of populations. Finally, to evaluate genetic differences between major Gulf regions, localities were grouped into upper (SF, SC, PP and PL) and central Gulf (BK, BG and HT) and an AMOVA was performed in Arlequin.

The effective population size (*N*_*e*_) was estimated for all pooled samples using the Linkage Disequilibrium (LD) method (Hill, 1981) using the random mating model, bias correction (Waples, 2006), and by the incorporation of missing data (Peel et al., 2013) as implemented in NeEstimator 2.0 (Do et al., 2014). As high levels of polymorphism in microsatellite loci having rare alleles can cause biased estimates of *N*_*e*_ (Waples, 2006), NeEstimator was used to screen out rare alleles with frequencies below the specific critical value of *P*_crit_ = 0.02 (Waples & Do, 2010). Upper and lower bounds of 95% confidence intervals (C.I.) were calculated for the *N*_*e*_ using the jackknife method in order to reduce the potential bias associated with the parametric confidence intervals for the LD method.

BayesAss 3 (Wilson & Rannala, 2003) was used to estimate contemporary gene flow between both regions. Three replicate runs were performed using 10,000,000 MCMC iterations, 2,000,000 burn-in iterations and sampling every 1,000 iterations. Then, the average migration rate between the upper-central Gulf and in the opposite direction was calculated.

A *mtCyt-b* fragment of 873 bp was sequenced in 199 individuals (Table 1). Sequences were aligned using the MUSCLE 3.8 application (Edgar, 2004) implemented in SeaView 4 (Gouy, Guindon & Gascuel, 2010). The number of haplotypes (*n*_*h*_), segregating sites (*S*), nucleotide (*π*) and haplotype (*h*) diversities, were calculated using Arlequin. A haplotype network was constructed using the software PopART 1.7 (http://popart.otago.ac.nz).

In order to select the best evolutionary model fitting the data, jModelTest 2.1.6 (Darriba et al., 2012) was used. A distance matrix based on the evolutionary model was used to estimate pairwise genetic differences between locations through Φ_*ST*_ in Arlequin. The existence of spatial genetic differences was examined through an AMOVA in Arlequin, pooling the samples into upper and central regions.

The mutational rate for *mtCyt-b* was calibrated using the sequences of *S. concolor* (*n* = 199) and *S. sierra* (*n* = 13) in BEAST 2 (Bouckaert et al., 2014). As priors we selected the calibrated Yule speciation model and all parameters set up as default values as suggested in Bouckaert et al. (2014), except for the divergence time for which we used a normal distribution with a mean of 2.3 million years. This mean corresponds to the estimated speciation time between *S. concolor* and *S. sierra* (Banford et al., 1999). A standard deviation of 0.5 M years, resulting in an estimate of 1.8% mutation per site per million years. A MCMC length of 100,000,000 genealogies with a burn-in period of 10,000,000 steps was used. BEAST 2 was used to construct a Bayesian skyline plot to assess changes in historical demographic size through time, using the estimated mutation rate of 1.8% divergence per site per million years (95% CI [0.8–3.0%]). Finally, Tracer 1.6 (Rambaut et al., 2014) was used for assessing effective sample size (EES) values for parameters and visualization.

Inferences about historical demography for the *S. concolor* population were obtained from the distribution of mismatches (Schneider & Excoffier, 1999). The observed distribution of mismatches was tested for adjustment to the demographic (Rogers & Harpending, 1992) and/or spatial expansion model (Excoffier, 2004). The parameter *τ*, Harpending’s raggedness index and the values for the sum-of-squares deviations (*SSD*) were estimated in Arlequin. The parameter *τ* was translated into years using the equation: *T* = *τ*∕2*μ*, where *μ* is the mutational rate of 1.8% per site per million years for the *mtCyt-b* and a generational time of four years for *S. concolor* was used (Valdovinos-Jacobo, 2006). Additionally, Fu’s *F* test (Fu, 1997) was performed in order to examine deviations to neutrality due to population expansion or selection.

**Table 2:** Genetic variability in *S. concolor mtCyt-b* sequences per location. Sequences were amplified from the following temporal samples: SF06, PP06, BK06 and BG05, and all the sequences pooled together into the entire Gulf of California (GC).

	Location							
	SF	SC	PP	PL	BK	BG	HT	GC
*n*	27	28	28	29	29	30	28	199
*S*	32	28	27	26	27	23	26	76
*n*_*h*_	21	19	21	19	19	18	19	78
*h*	0.966	0.952	0.955	0.931	0.924	0.947	0.947	0.940
*S*.*D*	0.025	0.026	0.030	0.035	0.041	0.026	0.030	0.012
*π*	0.0048	0.0050	0.0050	0.0043	0.0047	0.0051	0.0051	0.0048
*S*.*D*	0.0027	0.0028	0.0028	0.0025	0.0027	0.0029	0.0029	0.0027

**Notes.**

Sample size (*n*), segregating sites (*S*), number of haplotypes (*n*_*h*_), haplotype diversity (*h*), nucleotide diversity (*π*).

## Results

### Gene diversity

Analyses of Micro-Checker did not detect genotyping errors by stuttering or large allele dropout. However, null alleles were detected at locus *Sbr18* in SC, PP, BK and BG locations, and at locus *Sbr9* for PL and BK locations. However, significant *F*_*IS*_ due to heterozygosity deficit were obtained only in PP and BG locations at *Sbr18* locus (after Bonferroni correction; see Table S3). Additionally, this locus deviated significantly from Hardy-Weinberg expectations in the SC location after Bonferroni correction (Rice, 1989). As results for population divergence did not change by omitting this locus, it was decided to keep it for further analyses. No significant linkage disequilibrium was found, suggesting that the loci segregate independently.

The mean number of alleles per locus was 13.08 ± 2.14 with values ranging from 10.33 in the HT sample to 15.89 in the BK sample. The mean allelic richness obtained from a minimum sample number of 24 individuals was 10.48 ± 0.34 ranging from 9.89 (HT) to 10.92 (SC). The mean observed heterozygosity was 0.721 ± 0.024 and ranged from 0.675 (SC) to 0.744 (HT), whereas the mean expected heterozygosity was 0.736 ± 0.019 and ranged from 0.709 (SC) to 0.766 (BG). All diversity measures were very similar among the locations (see Table S3).

Sequences of 199 individuals resulted in 68 haplotypes containing 76 polymorphic sites (39 being singletons), 69 transitions and 9 transversions were obtained for the 873 bp fragment of *mtCyt-b*. The mean haplotype diversity was 0.940 ± 0.012 and ranged from 0.924 (BK) to 0.966 (SF), whereas the mean nucleotide diversity was 0.0048 ± 0.0027 ranging from 0.0043 (PL) to 0.0051 (BG) (Table 2).

### Genetic divergence

The correlation between *G*_*ST*_ and *H*_*S*_ estimates among the nine microsatellites was positive (*r* = 0.35), and not significant *P* = 0.356, implying that the use of *F*_*ST*_ is suitable for the succeeding analyses.

As the multilocus pairwise estimates of *F*_*ST*_ among the temporal collections were not significant (see Table S4), these were pooled into San Felipe (SF), Puerto Peñasco (PP), Bahia Kino (BK) and Bahía Guaymas (BG) for subsequent analyses.

The results of the pairwise multilocus *F*_*ST*_ among locations ranged from zero to the maximum value of 0.007 obtained between SC and BG, although no *F*_*ST*_ value showed significant differences after Bonferroni correction (Table 3) indicating temporal and spatial genetic homogeneity.

**Table 3:** Pairwise sample multilocus *F*_*ST*_ (below the diagonal) and Φ_*ST*_ (above the diagonal) estimates. Temporal collections were pooled into locations SF (SF06 and SF08), PP (PP06, PP07 and PP08), BK (BK05, BK06 and BK07), BG (BG05 and BG06). Significant probabilities for differences were considered after Bonferroni correction (*α* = 0.05∕21 = 0.0024).

	SF	SC	PP	PL	BK	BG	HT
SF	**–**	0.000	0.000	0.000	0.000	0.000	0.000
SC	0.005	**–**	0.000	0.000	0.000	0.000	0.000
PP	0.000	0.002	**–**	0.000	0.000	0.000	0.000
PL	0.001	0.001	0.000	**–**	0.006	0.000	0.012
BK	0.000	0.002	0.000	0.004	**–**	0.000	0.000
BG	0.001	0.007	0.000	0.002	0.000	**–**	0.000
HT	0.000	0.000	0.000	0.002	0.000	0.002	**–**

The most probable number of genetic populations obtained from the STRUCTURE analysis, either with or without admixture model, was *k* = 1 (mean *LnP*(*D*) =  − 15838.4 and −15838.2, respectively), suggesting no population structure (see Fig. S1). Accordingly, neither the AMOVA for temporal structure (*F*_*CT*_ = 0.00035, *P* = 0.268) nor the AMOVA for spatial structure showed no significant differences (*F*_*CT*_ = 0.00035, *P* = 0.286), being consistent with a panmictic *S. concolor* population in the GC.

Based on the distance matrix calculated with the HKY + I model (Hasegawa, Kishino & Yano, 1985) as estimated with jModelTest, the pairwise sample Φ_*ST*_ estimates varied from zero to 0.012 (PL-HT comparison) and none of the comparisons between locations exhibited significant genetic differences (Table 3). Similarly, the AMOVA showed no differences when grouping samples into upper and central Gulf (Φ_*CT*_ = 0.006, *P* = 0.061).

### *N*_*e*_ and genetic flow

The estimated contemporary effective population size (*N*_*e*_) as a single population using the Linkage Disequilibrium method (LD) and a *P*_*crit*_ = 0.02, was 3056.9. The upper and lower bound of 95% CI for the *N*_*e*_ estimates were (95% CI _jackknife_ = 1344.9-infinite).

The BayesAss results showed that migration rates are asymmetrical between regions, with the higher rates occurring from the CG to the UG $(\overline{m}=0.324)$ and the lower from the UG to the CG $(\overline{m}=0.024)$.

### Historical demography

**Figure 2:**
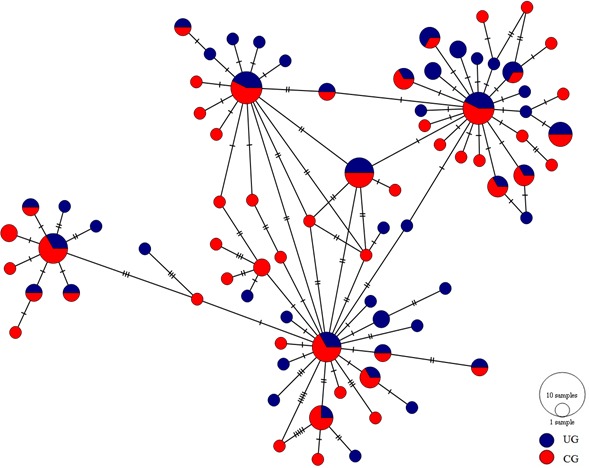
Haplotype network of the *mtCyt-b* of *S. concolor*. Circle size is proportional to the number of individuals representing a given haplotype. Dashes between haplotypes represent mutational steps. (UG = Upper Gulf, CG = Central Gulf).

The haplotype network showed four major distinguishable nodes, each one displaying a star-like topology where low frequency haplotypes are separated in most of the cases by a single mutation. Likewise, the haplotypes showed no spatial arrangement between the upper and central Gulf (Fig. 2).

**Figure 3:**
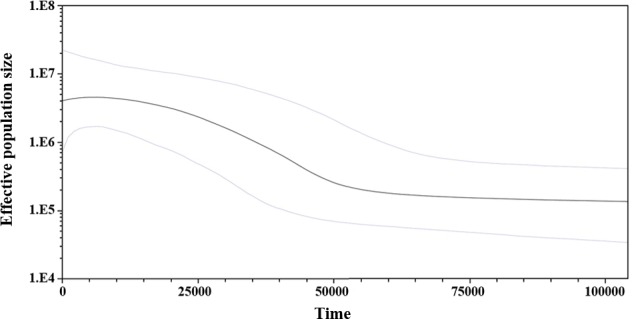
Bayesian skyline plot showing changes of effective population size (*N*_*e*_) through time based on *mtCyt-b* sequences of *S. concolor*. The bold line represents median estimate of *N*_*e*_ with a mutation rate of 1.8% M years^−1^. The area between blue lines corresponds to the 95% highest posterior density (HPD) region of *N*_*e*_. The *x*-axis is years before present and *y*-axis is on a log_10_ scale.

Considering the lack of genetic differences between locations and regions, an analysis of mismatches of *mtCyt-b* sequences for the whole *S. concolor* population was made, that showed a unimodal distribution characterized by a mode of four differences among sequences, suggesting the occurrence of population expansions. Additionally, Fu’s *F* value was negative and highly significant (*F* =  − 25.517, *P* < 0.001), supporting deviations from neutrality. Harpending’s raggedness index was low and non-significant (0.009; *P* = 0.890), as were the values for the sum-of-squares deviations (*SSD*), which did not deviate from the unimodal distribution (*P* = 0.685 and *P* = 0.534; respectively). The *τ* obtained under the demographic model was 3.5 and for the spatial model was 2.3. Based on these results obtained from the analysis of mismatches from the pairwise differences for all sequences (Rogers & Harpending, 1992) and using the estimated mutational rate of 1.8% for *mtCyt-b*, the population expansion (either demographic or spatial) occurred between 111,366 (95% CI [50,910–292,733]) and 73,183 (95% CI [31,819–178,185]) years before present (YBP). Additionally, and giving support to the previous results, the skyline plot (whose estimation is based on coalescent theory; Drummond et al., 2005), showed a slight population expansion between 50,000–20,000 YBP (Fig. 3).

## Discussion

### Gene diversity

Published studies have shown that microsatellites of pelagic species are highly polymorphic (Ruzzante, Taggart & Cook, 1996; Carlsson et al., 2004) especially for scombrids, and particularly for the *Scomberomorus* species (Renshaw et al., 2009; Lin et al., 2012). The observed heterozygosity $(\overline{Ho})$ was similar to those previously reported for another congeneric species, such as *S. cavalla* ($\overline{Ho}=0.711$; Broughton, Stewart & Gold, 2002 and $\overline{Ho}=0.604$; Gold, Pak & Devries, 2002); *S. brasiliensis* ($\overline{Ho}=0.673$; Gold et al., 2010), and *Scomberomorus semifasciatus* ($\overline{Ho}=0.623$; Broderick et al., 2011). The allelic richness and observed heterozygosity of all nine loci were similar across all locations suggesting the genetic homogeneity of the *S. concolor* population.

For the *mtCyt-b*, a high haplotype diversity (*h* = 0.940) was observed similar to those of congeneric species based on *mtDNA-CR* sequences (Pacific sierra *S. sierra h* = 0.997, Domínguez-López, Uribe-Alcocer & Díaz-Jaimes, 2010; the Japanese Spanish mackerel *S. niphonius h* = 0.996, Shui et al., 2009 and the narrow-barred Spanish mackerel *Scomberomorus commerson h* = 0.986, Hoolihan, Anandh & Van Herwerden, 2006). However, as expected for the *mtCyt-b* gene characterized by a lower mutational rate, a notably lower nucleotide diversity (*π* = 0.0048) was observed than reported for the *mtDNA-CR* of other *Scomberomorus* species (*S. sierra π* = 0.0195, Domínguez-López, Uribe-Alcocer & Díaz-Jaimes, 2010; *S. niphonius π* = 0.0236, Shui et al., 2009 and *S. commerson π* = 0.0380, Hoolihan, Anandh & Van Herwerden, 2006). All these studies have reported genetic homogeneity for distant populations where high population size and gene flow have been argued to explain the lack of population divergence. This has been concluded also for other large pelagic fish species as the yellowfin tuna *Thunnus albacares* (*h* = 0.997; *π* = 0.0350; Ely et al., 2005), the skipjack tuna *Katsuwonus pelamis* (*h* = 0.999; *π* = 0.0840; Ely et al., 2005). The high genetic variation observed in *S. concolor* using both *mtDNA* and nuclear DNA markers, seems to be congruent with high gene flow and a large population size (Avise, Neigel & Arnold, 1984; Graves, 1998; Díaz-Jaimes et al., 2010) (for further discussion see below).

### Population genetic structure and gene flow

Most marine pelagic fish species show low levels of genetic differentiation due mainly to significant gene flow as consequence of their high dispersal capability during planktonic egg, larval or adult stages (Grant & Bowen, 1998). Furthermore, by considering that a small number of migrants per generation are sufficient to eliminate genetic differentiation and that marine species usually have high fecundities, it is usual to find low levels of differentiation (Waples, 1998). *Scomberomorus concolor* displays seasonal movements between the GC regions, so the absence of differentiation exhibited in both microsatellite data and *mtCyt-b* sequences can be explained by its migratory behavior. Both markers showed genetic homogeneity among temporal and spatial samples from the two regions of the GC. Neither the pairwise multilocus *F*_*ST*_ nor the pairwise Φ_*ST*_ revealed differences between localities, supporting the existence of a single homogeneous population in the GC. Moreover, the allelic richness and the observed heterozygosities are notably similar among the localities of both Gulf regions, and all the microsatellite loci for the *S. concolor* population are in Hardy–Weinberg equilibrium, with the exception of the locus *Sn26* (see Table S3). In addition, no alteration in these estimates was detected when considering all samples as a single population, lending further support to the genetic homogeneity observed. The AMOVA results for both, microsatellites and *mtCyt-b*, showed no significant genetic differences between the locations grouped into the upper and central portions of the GC nor in any other hierarchical level. This supports the results obtained for the *mtDNA-CR* (Domínguez-López et al., 2015) in which the authors did not find any difference between the GC regions. Hence, based on evidence of nuclear and mitochondrial information, we can confirm the existence of a panmictic population of *S. concolor* in the GC. This was also supported by the haplotype network (Fig. 2), which did not show any consistent geographical distribution of haplotypes between regions of the GC: there are four highly frequent haplotypes distributed in both the upper and the central Gulf, each one derived in a star-shape fashion suggesting multiple expansion events in the relatively recent past (Slatkin & Hudson, 1991).

The absence of genetic differences between samples of *S. concolor* in the GC and also in other congeneric species (e.g., *Scomberomorus maculatus*, Buonaccorsi, Starkey & Graves, 2001; *S. commerson*, Hoolihan, Anandh & van Herwerden, 2006; *S. niphonius*, Shui et al., 2009) is consistent with the seasonal migrations that usually exhibit the members of Scombridae in response to changes in SST and food availability.

As cold water temperatures predominate in the upper Gulf during winter, *S concolor* moves to the central area where favorable conditions and food abundance prevail as resulted of high productive coastal upwelling areas in the central Gulf (Álvarez-Borrego, 2010). For this reason, this area has been considered a feeding ground for *S. concolor*. In contrast, during summer, *S. concolor* females with developing and ripe gonads move from the central to the upper Gulf where the warmer water and the high diversity and abundance of the fish larvae (Hidalgo-González & álvarez Borrego, 2001; Sánchez-Velasco et al., 2012) provides favorable conditions for spawning and recruitment. Therefore, *S. concolor* larvae may remain confined in the upper Gulf for growing.

Even though no tagging studies exist for assessing *S. concolor* movements between GC regions, Valdovinos-Jacobo (2006) has supported these seasonal migratory movements between the upper and central regions based on age structure and gonadic maturation of individuals. In the upper Gulf, during the summer season (May through June) a high number of large gravid *S. concolor* individuals ready to spawn are found, in coincidence with significant concentrations of *Scomberomorus spp.* larvae (Moser et al., 1973). By September, individuals with spent gonads are commonly found moving southward to the feeding grounds in the central Gulf suggesting the end of the spawning season (Valdovinos-Jacobo, 2006). This is consistent with our contemporary gene flow estimates which showed an asymmetrical movement of individuals, being the migration rate from the central to the upper region higher than the migration rate in the opposite direction, and in consequence implying migration of *S. concolor* individuals between the upper and central Gulf regions.

Based on the seasonal movements related with spawning and feeding and the findings presented in this study, it is possible to conclude that *S. concolor* comprises one single genetic transient population and that most of its young and adult members distribute seasonally in both Gulf regions, while the youngest individuals remain in the upper part for growth and posterior recruitment (Valdovinos-Jacobo, 2006).

### Effective population size and population history

The contemporary effective population size estimated for the *S. concolor* population with the Linkage Disequilibrium method (LD) was 3056.9. This large population size is similar to that observed in other pelagic fishes (Graves, 1998; Díaz-Jaimes et al., 2010) and is influenced by the high fecundity of the scombrid species. Although there are no fecundity estimates for *S. concolor*, it is likely that females possess a high reproductive potential, similar to other congeneric species such as *Scomberomorus guttatus* (Devaraj, 1987) and *S. cavalla* (Finucane et al., 1986), in which the fecundity of females has been estimated to be several hundreds of thousands eggs (e.g., 100,000–900,000 eggs). The effective population size may indicate whether a population can maintain adequate genetic variance for adaptive evolution (Tallmon et al., 2010) and is an important parameter in the management of populations of endangered species (Wang, 2005). Palstra & Ruzzante (2008) suggested that *N*_*e*_ values greater than 1000 are required to prevent deleterious allele accumulation. In our case the estimated ${\hat {N}}_{e}=3056.9$ is above the suggested threshold and do not correspond with a population of *S. concolor* at risk of collapse. However, interpreting estimates of *N*_*e*_ in fisheries management results controversial because of the marked differences between census population sizes and those based on genetic data (Hauser & Carvalho, 2008).

The results of the mismatch analyses support the occurrence in the past, of a reduction in the *S. concolor* population followed by an expansion according with the sudden expansion model (Rogers & Harpending, 1992), and/or the spatial expansion model (Excoffier, 2004). Using the estimated mutation rate of 1.8%, the population expansion either demographic or spatial, might have occurred between 73,000 and 111,000 YBP. The skyline plot is also coincident with the occurrence of a slight population expansion between 20,000 and 50,000 YBP. This estimation was coincident with the second expansion event detected through the mitochondrial control region by Domínguez-López et al. (2015).

The expansion event detected could have occurred during the interglacial period after the collapse of populations caused by glacial events in the Pleistocene. This finding is concordant with a decrease in the SST in the Gulf during glacial events 150,000 YBP that had a strong effect on other pelagic species living in the Gulf such as the Pacific sardine and the anchovy (Díaz-Viloria, Sánchez-Velasco & Pérez-Enríquez, 2012).

## Conclusions

*Scomberomorus concolor* comprises a single panmictic population in the Gulf of California, as was proved by the mitochondrial and nuclear markers. The population performs seasonal movements between the upper and central Gulf according with feeding and spawning activities resulting in temporal abundances in its main distributional areas. Our results provide relevant information useful for the design of management plans for the fishery as the existence of a single stock of *S. concolor* in the Gulf of California supports the implementation of separate management regimes for both, *S. concolor* and *S. sierra* coexisting in the central part of the Gulf. Likewise, the highly significant gene flow between both GC regions supports the seasonal migratory pattern that needs confirmation through tagging data, in order to implement a special protective regime for the unit present in the upper Gulf during the spawning season.

Finally, the estimated contemporary effective population size of this important commercial resource suggests that the population of *S. concolor* is genetically healthy. In terms of fisheries management, establishing separate catch quotes for *S. concolor* may help to maintain the genetic diversity shaped by the evolutionary history of the species during colonization and confinement in the Gulf of California.

##  Supplemental Information

10.7717/peerj.2583/supp-1 Figure S1 Summary results from the STRUCTURE analysis for *k* = 1

10.7717/peerj.2583/supp-2 Table S1 *Scomberomorus concolor* individuals collected by artisanal fisheries **Sample code.** The codes are comprised by the location and year of collection (San Felipe 2006 = SF06, San Felipe 2008 = SF08, Santa Clara 2006 = SC06, Puerto Peñasco 2006 = PP06, Puerto Peñasco 2007 = PP07, Puerto Peñasco 2008 = PP08, Puerto Puerto Libertad 2006 = PL06, Bahía Kino 2005 = BK05, Bahía Kino 2006 = BK06, Bahía Kino 2007 = BK07, Bahía Guaymas 2005 = BG05, Bahía Guaymas 2006 = BG06, and Huatabampo 2005 = HT05) following by the number of each individual. For example: SF0601 corresponds to the individual number 1, sampled in the location of San Felipe in 2006. **Total sample size:** *n* = 482. **Sample size by location:** SF06 = 37, SF08 = 32, SC06 = 29, PP06; *n* = 29, PP07 = 15, PP08 = 32, PL = 30, BK05 = 50, BK06 = 60, BK07 = 31, BG05 = 70, BG06 = 39 and HT = 28. * Month not available.

10.7717/peerj.2583/supp-3 Table S2 Summary statistics for nine microsatellite loci amplified for *S. concolor* for each location and for the entire population in the GC

10.7717/peerj.2583/supp-4 Table S3 Summary statistics for microsatellites Summary statistics for nine microsatellite loci amplified for *S. concolor* for each location and for the entire population in the GC.

10.7717/peerj.2583/supp-5 Table S4 Results of *F*_*ST*_ pairwise sample comparisons over temporal collections Results of *F*_*ST*_ pairwise sample comparisons over temporal collections. (A) San Felipe (SF) collections. (B) Puerto Peñasco (PP) comparisons. (C) Bahía Kino (BK) comparisons and (D) Bahía Guaymas (BG) comparisons. None *F*_*ST*_ value showed significant differences (*p* < 0.05).

## Additional Information and Declarations

### DNA Deposition

The following information was supplied regarding the deposition of DNA sequences: GenBank accession numbers:  KX462440–KX462527

### Data Availability

The following information was supplied regarding data availability: Magallon E. 2016. Genotypes file.txt. Figshare:
https://dx.doi.org/10.6084/m9.figshare.3443129.v1.
