# Supplementary material for: Spatial and temporal genetic homogeneity of the Monterey Spanish mackerel, Scomberomorus concolor, in the Gulf of California
Source: PeerJ. 2016 Oct 25;4:e2583. doi: 10.7717/peerj.2583 (PMC5088583; doi:10.7717/peerj.2583)
Supplement: Figure S1 [file peerj-04-2583-s001.pdf]

Mean posterior probability (LnP(D)) values from *k*=1 to *k=7* from the STRUCTURE analysis. The most probable number of genetic populations is *k*=1 with **(A)** or without admixture model **(B)**.

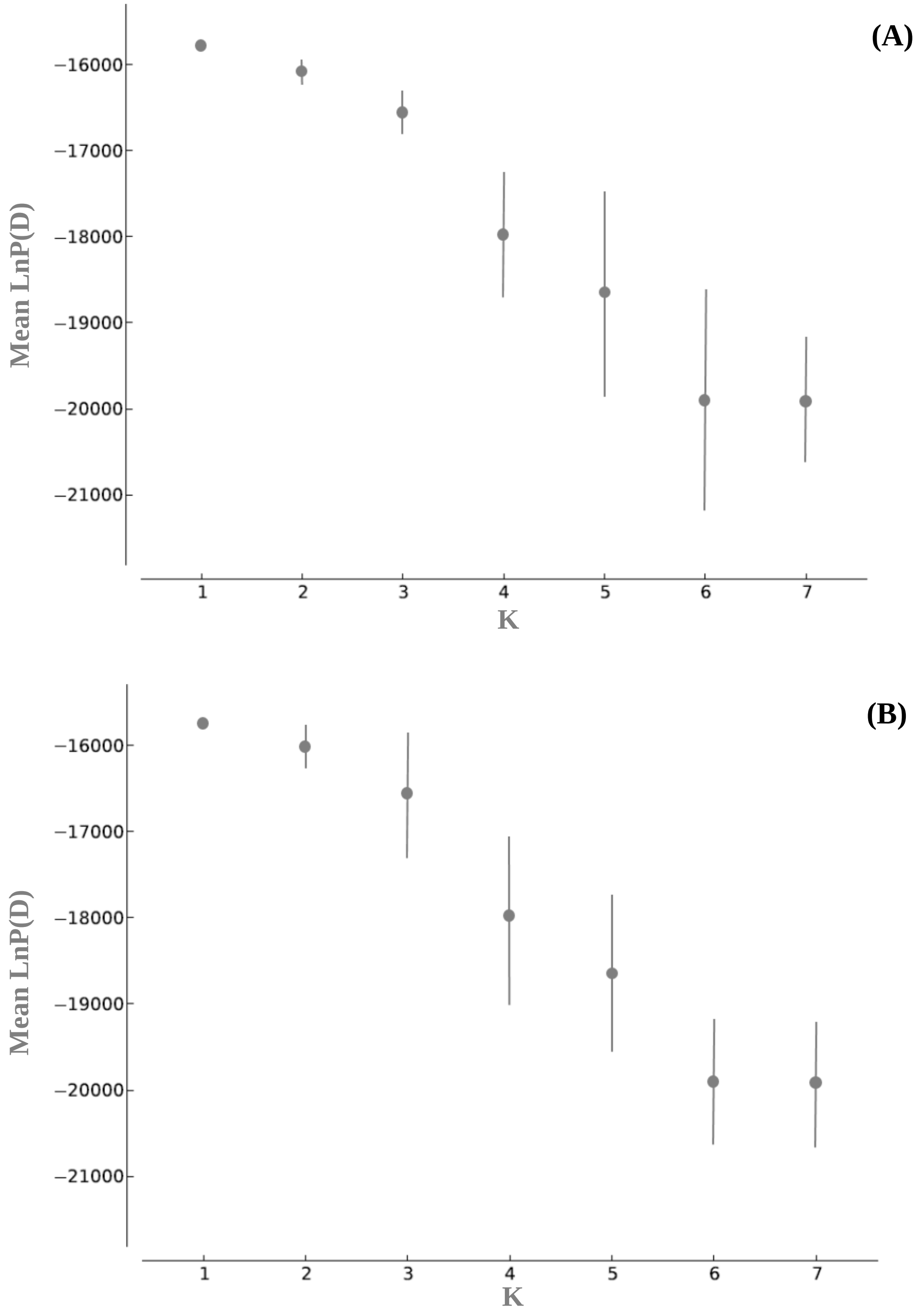

Mean LnP(D) values are represented by circles and bars represent the standard deviation (s.d).
