# Supplementary material for: Spatial and temporal genetic homogeneity of the Monterey Spanish mackerel, Scomberomorus concolor, in the Gulf of California
Source: PeerJ. 2016 Oct 25;4:e2583. doi: 10.7717/peerj.2583 (PMC5088583; doi:10.7717/peerj.2583)
Supplement: Table S1 — Sample code. The codes are comprised by the location and year of collection (San Felipe 2006 = SF06, San Felipe 2008 = SF08, Santa Clara 2006 = SC06, Puerto Peñasco 2006 = PP06, Puerto Peñasco 2007 = PP07, Puerto Peñasco 2008 = PP08, Puerto Puerto Libertad 2006 = PL06, Bahía Kino 2005 = BK05, Bahía Kino 2006 = BK06, Bahía Kino 2007 = BK07, Bahía Guaymas 2005 = BG05, Bahía Guaymas 2006 = BG06, and Huatabampo 2005 = HT05) following by the number of each individual. For example: SF0601 corresponds to the individual number 1, sampled in the location of San Felipe in 2006. Total sample size: n = 482. Sample size by location: SF06 = 37, SF08 = 32, SC06 = 29, PP06; n = 29, PP07 = 15, PP08 = 32, PL = 30, BK05 = 50, BK06 = 60, BK07 = 31, BG05 = 70, BG06 = 39 and HT = 28. * Month not available. [file peerj-04-2583-s002.docx]

*Scomberomorus concolor* individuals collected by artisanal fisheries.

**Sample code.** The codes are comprised by the location and year of collection (San Felipe 2006=SF06, San Felipe 2008=SF08, Santa Clara 2006=SC06, Puerto Peñasco 2006=PP06, Puerto Peñasco 2007=PP07, Puerto Peñasco 2008=PP08, Puerto Puerto Libertad 2006=PL06, Bahía Kino 2005=BK05, Bahía Kino 2006=BK06, Bahía Kino 2007=BK07, Bahía Guaymas 2005=BG05, Bahía Guaymas 2006= BG06, and Huatabampo 2005=HT05) following by the number of each individual. For example: SF0601 corresponds to the individual number 1, sampled in the location of San Felipe in 2006.

**Total sample size:** n=482.

**Sample size by location:** SF06=37, SF08=32, SC06=29, PP06; n=29, PP07=15, PP08=32, PL=30, BK05=50, BK06=60, BK07=31, BG05=70, BG06=39 and HT=28.

* Month not available.

| **Sample code** | **Location** | **Date of collection** | **Latitude** | **Longitude** |
| --- | --- | --- | --- | --- |
| SF0601 | San Felipe | 2006* | 31º01'39 N | 114º50'07 W |
| SF0602 | San Felipe | 2006* | 31º01'39 N | 114º50'07 W |
| SF0603 | San Felipe | 2006* | 31º01'39 N | 114º50'07 W |
| SF0604 | San Felipe | 2006* | 31º01'39 N | 114º50'07 W |
| SF0605 | San Felipe | 2006* | 31º01'39 N | 114º50'07 W |
| SF0606 | San Felipe | 2006* | 31º01'39 N | 114º50'07 W |
| SF0607 | San Felipe | 2006* | 31º01'39 N | 114º50'07 W |
| SF0608 | San Felipe | 2006* | 31º01'39 N | 114º50'07 W |
| SF0609 | San Felipe | 2006* | 31º01'39 N | 114º50'07 W |
| SF0610 | San Felipe | 2006* | 31º01'39 N | 114º50'07 W |
| SF0611 | San Felipe | 2006* | 31º01'39 N | 114º50'07 W |
| SF0612 | San Felipe | 2006* | 31º01'39 N | 114º50'07 W |
| SF0613 | San Felipe | 2006* | 31º01'39 N | 114º50'07 W |
| SF0614 | San Felipe | 2006* | 31º01'39 N | 114º50'07 W |
| SF0615 | San Felipe | 2006* | 31º01'39 N | 114º50'07 W |
| SF0616 | San Felipe | 2006* | 31º01'39 N | 114º50'07 W |
| SF0617 | San Felipe | 2006* | 31º01'39 N | 114º50'07 W |
| SF0618 | San Felipe | 2006* | 31º01'39 N | 114º50'07 W |
| SF0619 | San Felipe | 2006* | 31º01'39 N | 114º50'07 W |
| SF0620 | San Felipe | 2006* | 31º01'39 N | 114º50'07 W |
| SF0621 | San Felipe | 2006* | 31º01'39 N | 114º50'07 W |
| SF0622 | San Felipe | 2006* | 31º01'39 N | 114º50'07 W |
| SF0623 | San Felipe | 2006* | 31º01'39 N | 114º50'07 W |
| SF0624 | San Felipe | 2006* | 31º01'39 N | 114º50'07 W |
| SF0625 | San Felipe | 2006* | 31º01'39 N | 114º50'07 W |
| SF0626 | San Felipe | 2006* | 31º01'39 N | 114º50'07 W |
| SF0627 | San Felipe | 2006* | 31º01'39 N | 114º50'07 W |
| SF0628 | San Felipe | 2006* | 31º01'39 N | 114º50'07 W |
| SF0629 | San Felipe | 2006* | 31º01'39 N | 114º50'07 W |
| SF0630 | San Felipe | 2006* | 31º01'39 N | 114º50'07 W |
| SF0631 | San Felipe | 2006* | 31º01'39 N | 114º50'07 W |
| SF0632 | San Felipe | 2006* | 31º01'39 N | 114º50'07 W |
| SF0633 | San Felipe | 2006* | 31º01'39 N | 114º50'07 W |
| SF0634 | San Felipe | 2006* | 31º01'39 N | 114º50'07 W |
| SF0635 | San Felipe | 2006* | 31º01'39 N | 114º50'07 W |
| SF0636 | San Felipe | 2006* | 31º01'39 N | 114º50'07 W |
| SF0637 | San Felipe | 2006* | 31º01'39 N | 114º50'07 W |
| SF0801 | San Felipe | May 2008 | 31º01'39 N | 114º50'07 W |
| SF0802 | San Felipe | May 2008 | 31º01'39 N | 114º50'07 W |
| SF0803 | San Felipe | May 2008 | 31º01'39 N | 114º50'07 W |
| SF0804 | San Felipe | May 2008 | 31º01'39 N | 114º50'07 W |
| SF0805 | San Felipe | May 2008 | 31º01'39 N | 114º50'07 W |
| SF0806 | San Felipe | May 2008 | 31º01'39 N | 114º50'07 W |
| SF0807 | San Felipe | May 2008 | 31º01'39 N | 114º50'07 W |
| SF0808 | San Felipe | May 2008 | 31º01'39 N | 114º50'07 W |
| SF0809 | San Felipe | May 2008 | 31º01'39 N | 114º50'07 W |
| SF0810 | San Felipe | May 2008 | 31º01'39 N | 114º50'07 W |
| SF0811 | San Felipe | May 2008 | 31º01'39 N | 114º50'07 W |
| SF0812 | San Felipe | May 2008 | 31º01'39 N | 114º50'07 W |
| SF0813 | San Felipe | May 2008 | 31º01'39 N | 114º50'07 W |
| SF0814 | San Felipe | May 2008 | 31º01'39 N | 114º50'07 W |
| SF0815 | San Felipe | May 2008 | 31º01'39 N | 114º50'07 W |
| SF0816 | San Felipe | May 2008 | 31º01'39 N | 114º50'07 W |
| SF0817 | San Felipe | May 2008 | 31º01'39 N | 114º50'07 W |
| SF0818 | San Felipe | May 2008 | 31º01'39 N | 114º50'07 W |
| SF0819 | San Felipe | May 2008 | 31º01'39 N | 114º50'07 W |
| SF0820 | San Felipe | May 2008 | 31º01'39 N | 114º50'07 W |
| SF0821 | San Felipe | May 2008 | 31º01'39 N | 114º50'07 W |
| SF0822 | San Felipe | May 2008 | 31º01'39 N | 114º50'07 W |
| SF0823 | San Felipe | May 2008 | 31º01'39 N | 114º50'07 W |
| SF0824 | San Felipe | May 2008 | 31º01'39 N | 114º50'07 W |
| SF0825 | San Felipe | May 2008 | 31º01'39 N | 114º50'07 W |
| SF0826 | San Felipe | May 2008 | 31º01'39 N | 114º50'07 W |
| SF0827 | San Felipe | May 2008 | 31º01'39 N | 114º50'07 W |
| SF0828 | San Felipe | May 2008 | 31º01'39 N | 114º50'07 W |
| SF0829 | San Felipe | May 2008 | 31º01'39 N | 114º50'07 W |
| SF0830 | San Felipe | May 2008 | 31º01'39 N | 114º50'07 W |
| SF0831 | San Felipe | May 2008 | 31º01'39 N | 114º50'07 W |
| SF0832 | San Felipe | May 2008 | 31º01'39 N | 114º50'07 W |
| SC0601 | Santa Clara | April 2006 | 31º41'12 N | 114º29'59 W |
| SC0602 | Santa Clara | April 2006 | 31º41'12 N | 114º29'59 W |
| SC0603 | Santa Clara | April 2006 | 31º41'12 N | 114º29'59 W |
| SC0604 | Santa Clara | April 2006 | 31º41'12 N | 114º29'59 W |
| SC0605 | Santa Clara | April 2006 | 31º41'12 N | 114º29'59 W |
| SC0606 | Santa Clara | April 2006 | 31º41'12 N | 114º29'59 W |
| SC0607 | Santa Clara | April 2006 | 31º41'12 N | 114º29'59 W |
| SC0608 | Santa Clara | April 2006 | 31º41'12 N | 114º29'59 W |
| SC0609 | Santa Clara | April 2006 | 31º41'12 N | 114º29'59 W |
| SC0610 | Santa Clara | April 2006 | 31º41'12 N | 114º29'59 W |
| SC0611 | Santa Clara | April 2006 | 31º41'12 N | 114º29'59 W |
| SC0612 | Santa Clara | April 2006 | 31º41'12 N | 114º29'59 W |
| SC0613 | Santa Clara | April 2006 | 31º41'12 N | 114º29'59 W |
| SC0614 | Santa Clara | April 2006 | 31º41'12 N | 114º29'59 W |
| SC0615 | Santa Clara | April 2006 | 31º41'12 N | 114º29'59 W |
| SC0616 | Santa Clara | April 2006 | 31º41'12 N | 114º29'59 W |
| SC0617 | Santa Clara | April 2006 | 31º41'12 N | 114º29'59 W |
| SC0618 | Santa Clara | April 2006 | 31º41'12 N | 114º29'59 W |
| SC0619 | Santa Clara | April 2006 | 31º41'12 N | 114º29'59 W |
| SC0620 | Santa Clara | April 2006 | 31º41'12 N | 114º29'59 W |
| SC0621 | Santa Clara | April 2006 | 31º41'12 N | 114º29'59 W |
| SC0622 | Santa Clara | April 2006 | 31º41'12 N | 114º29'59 W |
| SC0623 | Santa Clara | April 2006 | 31º41'12 N | 114º29'59 W |
| SC0624 | Santa Clara | April 2006 | 31º41'12 N | 114º29'59 W |
| SC0625 | Santa Clara | April 2006 | 31º41'12 N | 114º29'59 W |
| SC0626 | Santa Clara | April 2006 | 31º41'12 N | 114º29'59 W |
| SC0627 | Santa Clara | April 2006 | 31º41'12 N | 114º29'59 W |
| SC0628 | Santa Clara | April 2006 | 31º41'12 N | 114º29'59 W |
| SC0629 | Santa Clara | April 2006 | 31º41'12 N | 114º29'59 W |
| PP0601 | Puerto Peñasco | September 2006 | 31º19'00 N | 113º32'13 W |
| PP0602 | Puerto Peñasco | September 2006 | 31º19'00 N | 113º32'13 W |
| PP0603 | Puerto Peñasco | September 2006 | 31º19'00 N | 113º32'13 W |
| PP0604 | Puerto Peñasco | September 2006 | 31º19'00 N | 113º32'13 W |
| PP0605 | Puerto Peñasco | September 2006 | 31º19'00 N | 113º32'13 W |
| PP0606 | Puerto Peñasco | September 2006 | 31º19'00 N | 113º32'13 W |
| PP0607 | Puerto Peñasco | September 2006 | 31º19'00 N | 113º32'13 W |
| PP0608 | Puerto Peñasco | September 2006 | 31º19'00 N | 113º32'13 W |
| PP0609 | Puerto Peñasco | September 2006 | 31º19'00 N | 113º32'13 W |
| PP0610 | Puerto Peñasco | September 2006 | 31º19'00 N | 113º32'13 W |
| PP0611 | Puerto Peñasco | September 2006 | 31º19'00 N | 113º32'13 W |
| PP0612 | Puerto Peñasco | September 2006 | 31º19'00 N | 113º32'13 W |
| PP0613 | Puerto Peñasco | September 2006 | 31º19'00 N | 113º32'13 W |
| PP0614 | Puerto Peñasco | September 2006 | 31º19'00 N | 113º32'13 W |
| PP0615 | Puerto Peñasco | September 2006 | 31º19'00 N | 113º32'13 W |
| PP0616 | Puerto Peñasco | September 2006 | 31º19'00 N | 113º32'13 W |
| PP0617 | Puerto Peñasco | September 2006 | 31º19'00 N | 113º32'13 W |
| PP0618 | Puerto Peñasco | September 2006 | 31º19'00 N | 113º32'13 W |
| PP0619 | Puerto Peñasco | September 2006 | 31º19'00 N | 113º32'13 W |
| PP0620 | Puerto Peñasco | September 2006 | 31º19'00 N | 113º32'13 W |
| PP0621 | Puerto Peñasco | September 2006 | 31º19'00 N | 113º32'13 W |
| PP0622 | Puerto Peñasco | September 2006 | 31º19'00 N | 113º32'13 W |
| PP0623 | Puerto Peñasco | September 2006 | 31º19'00 N | 113º32'13 W |
| PP0624 | Puerto Peñasco | September 2006 | 31º19'00 N | 113º32'13 W |
| PP0625 | Puerto Peñasco | September 2006 | 31º19'00 N | 113º32'13 W |
| PP0626 | Puerto Peñasco | September 2006 | 31º19'00 N | 113º32'13 W |
| PP0627 | Puerto Peñasco | September 2006 | 31º19'00 N | 113º32'13 W |
| PP0628 | Puerto Peñasco | September 2006 | 31º19'00 N | 113º32'13 W |
| PP0629 | Puerto Peñasco | September 2006 | 31º19'00 N | 113º32'13 W |
| PP0701 | Puerto Peñasco | August 2007 | 31º19'00 N | 113º32'13 W |
| PP0702 | Puerto Peñasco | August 2007 | 31º19'00 N | 113º32'13 W |
| PP0703 | Puerto Peñasco | August 2007 | 31º19'00 N | 113º32'13 W |
| PP0704 | Puerto Peñasco | August 2007 | 31º19'00 N | 113º32'13 W |
| PP0705 | Puerto Peñasco | August 2007 | 31º19'00 N | 113º32'13 W |
| PP0706 | Puerto Peñasco | August 2007 | 31º19'00 N | 113º32'13 W |
| PP0707 | Puerto Peñasco | August 2007 | 31º19'00 N | 113º32'13 W |
| PP0708 | Puerto Peñasco | August 2007 | 31º19'00 N | 113º32'13 W |
| PP0709 | Puerto Peñasco | August 2007 | 31º19'00 N | 113º32'13 W |
| PP0710 | Puerto Peñasco | August 2007 | 31º19'00 N | 113º32'13 W |
| PP0711 | Puerto Peñasco | August 2007 | 31º19'00 N | 113º32'13 W |
| PP0712 | Puerto Peñasco | August 2007 | 31º19'00 N | 113º32'13 W |
| PP0713 | Puerto Peñasco | August 2007 | 31º19'00 N | 113º32'13 W |
| PP0714 | Puerto Peñasco | August 2007 | 31º19'00 N | 113º32'13 W |
| PP0715 | Puerto Peñasco | August 2007 | 31º19'00 N | 113º32'13 W |
| PP0801 | Puerto Peñasco | April 2008 | 31º19'00 N | 113º32'13 W |
| PP0802 | Puerto Peñasco | April 2008 | 31º19'00 N | 113º32'13 W |
| PP0803 | Puerto Peñasco | April 2008 | 31º19'00 N | 113º32'13 W |
| PP0804 | Puerto Peñasco | April 2008 | 31º19'00 N | 113º32'13 W |
| PP0805 | Puerto Peñasco | April 2008 | 31º19'00 N | 113º32'13 W |
| PP0806 | Puerto Peñasco | April 2008 | 31º19'00 N | 113º32'13 W |
| PP0807 | Puerto Peñasco | April 2008 | 31º19'00 N | 113º32'13 W |
| PP0808 | Puerto Peñasco | April 2008 | 31º19'00 N | 113º32'13 W |
| PP0809 | Puerto Peñasco | April 2008 | 31º19'00 N | 113º32'13 W |
| PP0810 | Puerto Peñasco | April 2008 | 31º19'00 N | 113º32'13 W |
| PP0811 | Puerto Peñasco | April 2008 | 31º19'00 N | 113º32'13 W |
| PP0812 | Puerto Peñasco | April 2008 | 31º19'00 N | 113º32'13 W |
| PP0813 | Puerto Peñasco | April 2008 | 31º19'00 N | 113º32'13 W |
| PP0814 | Puerto Peñasco | April 2008 | 31º19'00 N | 113º32'13 W |
| PP0815 | Puerto Peñasco | April 2008 | 31º19'00 N | 113º32'13 W |
| PP0816 | Puerto Peñasco | April 2008 | 31º19'00 N | 113º32'13 W |
| PP0817 | Puerto Peñasco | April 2008 | 31º19'00 N | 113º32'13 W |
| PP0818 | Puerto Peñasco | April 2008 | 31º19'00 N | 113º32'13 W |
| PP0819 | Puerto Peñasco | April 2008 | 31º19'00 N | 113º32'13 W |
| PP0820 | Puerto Peñasco | April 2008 | 31º19'00 N | 113º32'13 W |
| PP0821 | Puerto Peñasco | April 2008 | 31º19'00 N | 113º32'13 W |
| PP0822 | Puerto Peñasco | April 2008 | 31º19'00 N | 113º32'13 W |
| PP0823 | Puerto Peñasco | April 2008 | 31º19'00 N | 113º32'13 W |
| PP0824 | Puerto Peñasco | April 2008 | 31º19'00 N | 113º32'13 W |
| PP0825 | Puerto Peñasco | April 2008 | 31º19'00 N | 113º32'13 W |
| PP0826 | Puerto Peñasco | April 2008 | 31º19'00 N | 113º32'13 W |
| PP0827 | Puerto Peñasco | April 2008 | 31º19'00 N | 113º32'13 W |
| PP0828 | Puerto Peñasco | April 2008 | 31º19'00 N | 113º32'13 W |
| PP0829 | Puerto Peñasco | April 2008 | 31º19'00 N | 113º32'13 W |
| PP0830 | Puerto Peñasco | April 2008 | 31º19'00 N | 113º32'13 W |
| PP0831 | Puerto Peñasco | April 2008 | 31º19'00 N | 113º32'13 W |
| PP0832 | Puerto Peñasco | April 2008 | 31º19'00 N | 113º32'13 W |
| PL0601 | Puerto Libertad | April 2006 | 29º54'15 N | 112º40'59 W |
| PL0602 | Puerto Libertad | April 2006 | 29º54'15 N | 112º40'59 W |
| PL0603 | Puerto Libertad | April 2006 | 29º54'15 N | 112º40'59 W |
| PL0604 | Puerto Libertad | April 2006 | 29º54'15 N | 112º40'59 W |
| PL0605 | Puerto Libertad | April 2006 | 29º54'15 N | 112º40'59 W |
| PL0606 | Puerto Libertad | April 2006 | 29º54'15 N | 112º40'59 W |
| PL0607 | Puerto Libertad | April 2006 | 29º54'15 N | 112º40'59 W |
| PL0608 | Puerto Libertad | April 2006 | 29º54'15 N | 112º40'59 W |
| PL0609 | Puerto Libertad | April 2006 | 29º54'15 N | 112º40'59 W |
| PL0610 | Puerto Libertad | April 2006 | 29º54'15 N | 112º40'59 W |
| PL0611 | Puerto Libertad | April 2006 | 29º54'15 N | 112º40'59 W |
| PL0612 | Puerto Libertad | April 2006 | 29º54'15 N | 112º40'59 W |
| PL0613 | Puerto Libertad | April 2006 | 29º54'15 N | 112º40'59 W |
| PL0614 | Puerto Libertad | April 2006 | 29º54'15 N | 112º40'59 W |
| PL0615 | Puerto Libertad | April 2006 | 29º54'15 N | 112º40'59 W |
| PL0616 | Puerto Libertad | April 2006 | 29º54'15 N | 112º40'59 W |
| PL0617 | Puerto Libertad | April 2006 | 29º54'15 N | 112º40'59 W |
| PL0618 | Puerto Libertad | April 2006 | 29º54'15 N | 112º40'59 W |
| PL0619 | Puerto Libertad | April 2006 | 29º54'15 N | 112º40'59 W |
| PL0620 | Puerto Libertad | April 2006 | 29º54'15 N | 112º40'59 W |
| PL0621 | Puerto Libertad | April 2006 | 29º54'15 N | 112º40'59 W |
| PL0622 | Puerto Libertad | April 2006 | 29º54'15 N | 112º40'59 W |
| PL0623 | Puerto Libertad | April 2006 | 29º54'15 N | 112º40'59 W |
| PL0624 | Puerto Libertad | April 2006 | 29º54'15 N | 112º40'59 W |
| PL0625 | Puerto Libertad | April 2006 | 29º54'15 N | 112º40'59 W |
| PL0626 | Puerto Libertad | April 2006 | 29º54'15 N | 112º40'59 W |
| PL0627 | Puerto Libertad | April 2006 | 29º54'15 N | 112º40'59 W |
| PL0628 | Puerto Libertad | April 2006 | 29º54'15 N | 112º40'59 W |
| PL0629 | Puerto Libertad | April 2006 | 29º54'15 N | 112º40'59 W |
| PL0630 | Puerto Libertad | April 2006 | 29º54'15 N | 112º40'59 W |
| BK0501 | Bahía Kino | April 2005 | 28º49'22 N | 111º56'27 W |
| BK0502 | Bahía Kino | April 2005 | 28º49'22 N | 111º56'27 W |
| BK0503 | Bahía Kino | April 2005 | 28º49'22 N | 111º56'27 W |
| BK0504 | Bahía Kino | April 2005 | 28º49'22 N | 111º56'27 W |
| BK0505 | Bahía Kino | April 2005 | 28º49'22 N | 111º56'27 W |
| BK0506 | Bahía Kino | April 2005 | 28º49'22 N | 111º56'27 W |
| BK0507 | Bahía Kino | April 2005 | 28º49'22 N | 111º56'27 W |
| BK0508 | Bahía Kino | April 2005 | 28º49'22 N | 111º56'27 W |
| BK0509 | Bahía Kino | April 2005 | 28º49'22 N | 111º56'27 W |
| BK0510 | Bahía Kino | April 2005 | 28º49'22 N | 111º56'27 W |
| BK0511 | Bahía Kino | April 2005 | 28º49'22 N | 111º56'27 W |
| BK0512 | Bahía Kino | April 2005 | 28º49'22 N | 111º56'27 W |
| BK0513 | Bahía Kino | April 2005 | 28º49'22 N | 111º56'27 W |
| BK0514 | Bahía Kino | April 2005 | 28º49'22 N | 111º56'27 W |
| BK0515 | Bahía Kino | April 2005 | 28º49'22 N | 111º56'27 W |
| BK0516 | Bahía Kino | April 2005 | 28º49'22 N | 111º56'27 W |
| BK0517 | Bahía Kino | April 2005 | 28º49'22 N | 111º56'27 W |
| BK0518 | Bahía Kino | April 2005 | 28º49'22 N | 111º56'27 W |
| BK0519 | Bahía Kino | April 2005 | 28º49'22 N | 111º56'27 W |
| BK0520 | Bahía Kino | April 2005 | 28º49'22 N | 111º56'27 W |
| BK0521 | Bahía Kino | April 2005 | 28º49'22 N | 111º56'27 W |
| BK0522 | Bahía Kino | April 2005 | 28º49'22 N | 111º56'27 W |
| BK0523 | Bahía Kino | April 2005 | 28º49'22 N | 111º56'27 W |
| BK0524 | Bahía Kino | April 2005 | 28º49'22 N | 111º56'27 W |
| BK0525 | Bahía Kino | April 2005 | 28º49'22 N | 111º56'27 W |
| BK0526 | Bahía Kino | April 2005 | 28º49'22 N | 111º56'27 W |
| BK0527 | Bahía Kino | April 2005 | 28º49'22 N | 111º56'27 W |
| BK0528 | Bahía Kino | April 2005 | 28º49'22 N | 111º56'27 W |
| BK0529 | Bahía Kino | April 2005 | 28º49'22 N | 111º56'27 W |
| BK0530 | Bahía Kino | April 2005 | 28º49'22 N | 111º56'27 W |
| BK0531 | Bahía Kino | April 2005 | 28º49'22 N | 111º56'27 W |
| BK0532 | Bahía Kino | April 2005 | 28º49'22 N | 111º56'27 W |
| BK0533 | Bahía Kino | April 2005 | 28º49'22 N | 111º56'27 W |
| BK0534 | Bahía Kino | April 2005 | 28º49'22 N | 111º56'27 W |
| BK0535 | Bahía Kino | April 2005 | 28º49'22 N | 111º56'27 W |
| BK0536 | Bahía Kino | April 2005 | 28º49'22 N | 111º56'27 W |
| BK0537 | Bahía Kino | April 2005 | 28º49'22 N | 111º56'27 W |
| BK0538 | Bahía Kino | April 2005 | 28º49'22 N | 111º56'27 W |
| BK0539 | Bahía Kino | April 2005 | 28º49'22 N | 111º56'27 W |
| BK0540 | Bahía Kino | April 2005 | 28º49'22 N | 111º56'27 W |
| BK0541 | Bahía Kino | April 2005 | 28º49'22 N | 111º56'27 W |
| BK0542 | Bahía Kino | April 2005 | 28º49'22 N | 111º56'27 W |
| BK0543 | Bahía Kino | April 2005 | 28º49'22 N | 111º56'27 W |
| BK0544 | Bahía Kino | April 2005 | 28º49'22 N | 111º56'27 W |
| BK0545 | Bahía Kino | April 2005 | 28º49'22 N | 111º56'27 W |
| BK0546 | Bahía Kino | April 2005 | 28º49'22 N | 111º56'27 W |
| BK0547 | Bahía Kino | April 2005 | 28º49'22 N | 111º56'27 W |
| BK0548 | Bahía Kino | April 2005 | 28º49'22 N | 111º56'27 W |
| BK0549 | Bahía Kino | April 2005 | 28º49'22 N | 111º56'27 W |
| BK0550 | Bahía Kino | April 2005 | 28º49'22 N | 111º56'27 W |
| BK0601 | Bahía Kino | April 2006 | 28º49'22 N | 111º56'27 W |
| BK0602 | Bahía Kino | April 2006 | 28º49'22 N | 111º56'27 W |
| BK0603 | Bahía Kino | April 2006 | 28º49'22 N | 111º56'27 W |
| BK0604 | Bahía Kino | April 2006 | 28º49'22 N | 111º56'27 W |
| BK0605 | Bahía Kino | April 2006 | 28º49'22 N | 111º56'27 W |
| BK0606 | Bahía Kino | April 2006 | 28º49'22 N | 111º56'27 W |
| BK0607 | Bahía Kino | April 2006 | 28º49'22 N | 111º56'27 W |
| BK0608 | Bahía Kino | April 2006 | 28º49'22 N | 111º56'27 W |
| BK0609 | Bahía Kino | April 2006 | 28º49'22 N | 111º56'27 W |
| BK0610 | Bahía Kino | April 2006 | 28º49'22 N | 111º56'27 W |
| BK0611 | Bahía Kino | April 2006 | 28º49'22 N | 111º56'27 W |
| BK0612 | Bahía Kino | April 2006 | 28º49'22 N | 111º56'27 W |
| BK0613 | Bahía Kino | April 2006 | 28º49'22 N | 111º56'27 W |
| BK0614 | Bahía Kino | April 2006 | 28º49'22 N | 111º56'27 W |
| BK0615 | Bahía Kino | April 2006 | 28º49'22 N | 111º56'27 W |
| BK0616 | Bahía Kino | April 2006 | 28º49'22 N | 111º56'27 W |
| BK0617 | Bahía Kino | April 2006 | 28º49'22 N | 111º56'27 W |
| BK0618 | Bahía Kino | April 2006 | 28º49'22 N | 111º56'27 W |
| BK0619 | Bahía Kino | April 2006 | 28º49'22 N | 111º56'27 W |
| BK0620 | Bahía Kino | April 2006 | 28º49'22 N | 111º56'27 W |
| BK0621 | Bahía Kino | April 2006 | 28º49'22 N | 111º56'27 W |
| BK0622 | Bahía Kino | April 2006 | 28º49'22 N | 111º56'27 W |
| BK0623 | Bahía Kino | April 2006 | 28º49'22 N | 111º56'27 W |
| BK0624 | Bahía Kino | April 2006 | 28º49'22 N | 111º56'27 W |
| BK0625 | Bahía Kino | April 2006 | 28º49'22 N | 111º56'27 W |
| BK0626 | Bahía Kino | April 2006 | 28º49'22 N | 111º56'27 W |
| BK0627 | Bahía Kino | April 2006 | 28º49'22 N | 111º56'27 W |
| BK0628 | Bahía Kino | April 2006 | 28º49'22 N | 111º56'27 W |
| BK0629 | Bahía Kino | April 2006 | 28º49'22 N | 111º56'27 W |
| BK0630 | Bahía Kino | April 2006 | 28º49'22 N | 111º56'27 W |
| BK0631 | Bahía Kino | April 2006 | 28º49'22 N | 111º56'27 W |
| BK0632 | Bahía Kino | April 2006 | 28º49'22 N | 111º56'27 W |
| BK0633 | Bahía Kino | April 2006 | 28º49'22 N | 111º56'27 W |
| BK0634 | Bahía Kino | April 2006 | 28º49'22 N | 111º56'27 W |
| BK0635 | Bahía Kino | April 2006 | 28º49'22 N | 111º56'27 W |
| BK0636 | Bahía Kino | April 2006 | 28º49'22 N | 111º56'27 W |
| BK0637 | Bahía Kino | April 2006 | 28º49'22 N | 111º56'27 W |
| BK0638 | Bahía Kino | April 2006 | 28º49'22 N | 111º56'27 W |
| BK0639 | Bahía Kino | April 2006 | 28º49'22 N | 111º56'27 W |
| BK0640 | Bahía Kino | April 2006 | 28º49'22 N | 111º56'27 W |
| BK0641 | Bahía Kino | April 2006 | 28º49'22 N | 111º56'27 W |
| BK0642 | Bahía Kino | April 2006 | 28º49'22 N | 111º56'27 W |
| BK0643 | Bahía Kino | April 2006 | 28º49'22 N | 111º56'27 W |
| BK0644 | Bahía Kino | April 2006 | 28º49'22 N | 111º56'27 W |
| BK0645 | Bahía Kino | April 2006 | 28º49'22 N | 111º56'27 W |
| BK0646 | Bahía Kino | April 2006 | 28º49'22 N | 111º56'27 W |
| BK0647 | Bahía Kino | April 2006 | 28º49'22 N | 111º56'27 W |
| BK0648 | Bahía Kino | April 2006 | 28º49'22 N | 111º56'27 W |
| BK0649 | Bahía Kino | April 2006 | 28º49'22 N | 111º56'27 W |
| BK0650 | Bahía Kino | April 2006 | 28º49'22 N | 111º56'27 W |
| BK0651 | Bahía Kino | April 2006 | 28º49'22 N | 111º56'27 W |
| BK0652 | Bahía Kino | April 2006 | 28º49'22 N | 111º56'27 W |
| BK0653 | Bahía Kino | April 2006 | 28º49'22 N | 111º56'27 W |
| BK0654 | Bahía Kino | April 2006 | 28º49'22 N | 111º56'27 W |
| BK0655 | Bahía Kino | April 2006 | 28º49'22 N | 111º56'27 W |
| BK0656 | Bahía Kino | April 2006 | 28º49'22 N | 111º56'27 W |
| BK0657 | Bahía Kino | April 2006 | 28º49'22 N | 111º56'27 W |
| BK0658 | Bahía Kino | April 2006 | 28º49'22 N | 111º56'27 W |
| BK0659 | Bahía Kino | April 2006 | 28º49'22 N | 111º56'27 W |
| BK0660 | Bahía Kino | April 2006 | 28º49'22 N | 111º56'27 W |
| BK0701 | Bahía Kino | February 2007 | 28º49'22 N | 111º56'27 W |
| BK0702 | Bahía Kino | February 2007 | 28º49'22 N | 111º56'27 W |
| BK0703 | Bahía Kino | February 2007 | 28º49'22 N | 111º56'27 W |
| BK0704 | Bahía Kino | February 2007 | 28º49'22 N | 111º56'27 W |
| BK0705 | Bahía Kino | February 2007 | 28º49'22 N | 111º56'27 W |
| BK0706 | Bahía Kino | February 2007 | 28º49'22 N | 111º56'27 W |
| BK0707 | Bahía Kino | February 2007 | 28º49'22 N | 111º56'27 W |
| BK0708 | Bahía Kino | February 2007 | 28º49'22 N | 111º56'27 W |
| BK0709 | Bahía Kino | February 2007 | 28º49'22 N | 111º56'27 W |
| BK0710 | Bahía Kino | February 2007 | 28º49'22 N | 111º56'27 W |
| BK0711 | Bahía Kino | February 2007 | 28º49'22 N | 111º56'27 W |
| BK0712 | Bahía Kino | February 2007 | 28º49'22 N | 111º56'27 W |
| BK0713 | Bahía Kino | February 2007 | 28º49'22 N | 111º56'27 W |
| BK0714 | Bahía Kino | February 2007 | 28º49'22 N | 111º56'27 W |
| BK0715 | Bahía Kino | February 2007 | 28º49'22 N | 111º56'27 W |
| BK0716 | Bahía Kino | February 2007 | 28º49'22 N | 111º56'27 W |
| BK0717 | Bahía Kino | February 2007 | 28º49'22 N | 111º56'27 W |
| BK0718 | Bahía Kino | February 2007 | 28º49'22 N | 111º56'27 W |
| BK0719 | Bahía Kino | February 2007 | 28º49'22 N | 111º56'27 W |
| BK0720 | Bahía Kino | February 2007 | 28º49'22 N | 111º56'27 W |
| BK0721 | Bahía Kino | February 2007 | 28º49'22 N | 111º56'27 W |
| BK0722 | Bahía Kino | February 2007 | 28º49'22 N | 111º56'27 W |
| BK0723 | Bahía Kino | February 2007 | 28º49'22 N | 111º56'27 W |
| BK0724 | Bahía Kino | February 2007 | 28º49'22 N | 111º56'27 W |
| BK0725 | Bahía Kino | February 2007 | 28º49'22 N | 111º56'27 W |
| BK0726 | Bahía Kino | February 2007 | 28º49'22 N | 111º56'27 W |
| BK0727 | Bahía Kino | February 2007 | 28º49'22 N | 111º56'27 W |
| BK0728 | Bahía Kino | February 2007 | 28º49'22 N | 111º56'27 W |
| BK0729 | Bahía Kino | February 2007 | 28º49'22 N | 111º56'27 W |
| BK0730 | Bahía Kino | February 2007 | 28º49'22 N | 111º56'27 W |
| BK0731 | Bahía Kino | February 2007 | 28º49'22 N | 111º56'27 W |
| BG0501 | Bahía Guaymas | March 2005 | 27º55'06 N | 110º53'56 W |
| BG0502 | Bahía Guaymas | March 2005 | 27º55'06 N | 110º53'56 W |
| BG0503 | Bahía Guaymas | March 2005 | 27º55'06 N | 110º53'56 W |
| BG0504 | Bahía Guaymas | March 2005 | 27º55'06 N | 110º53'56 W |
| BG0505 | Bahía Guaymas | March 2005 | 27º55'06 N | 110º53'56 W |
| BG0506 | Bahía Guaymas | March 2005 | 27º55'06 N | 110º53'56 W |
| BG0507 | Bahía Guaymas | March 2005 | 27º55'06 N | 110º53'56 W |
| BG0508 | Bahía Guaymas | March 2005 | 27º55'06 N | 110º53'56 W |
| BG0509 | Bahía Guaymas | March 2005 | 27º55'06 N | 110º53'56 W |
| BG0510 | Bahía Guaymas | March 2005 | 27º55'06 N | 110º53'56 W |
| BG0511 | Bahía Guaymas | March 2005 | 27º55'06 N | 110º53'56 W |
| BG0512 | Bahía Guaymas | March 2005 | 27º55'06 N | 110º53'56 W |
| BG0513 | Bahía Guaymas | March 2005 | 27º55'06 N | 110º53'56 W |
| BG0514 | Bahía Guaymas | March 2005 | 27º55'06 N | 110º53'56 W |
| BG0515 | Bahía Guaymas | March 2005 | 27º55'06 N | 110º53'56 W |
| BG0516 | Bahía Guaymas | March 2005 | 27º55'06 N | 110º53'56 W |
| BG0517 | Bahía Guaymas | March 2005 | 27º55'06 N | 110º53'56 W |
| BG0518 | Bahía Guaymas | March 2005 | 27º55'06 N | 110º53'56 W |
| BG0519 | Bahía Guaymas | March 2005 | 27º55'06 N | 110º53'56 W |
| BG0520 | Bahía Guaymas | March 2005 | 27º55'06 N | 110º53'56 W |
| BG0521 | Bahía Guaymas | March 2005 | 27º55'06 N | 110º53'56 W |
| BG0522 | Bahía Guaymas | March 2005 | 27º55'06 N | 110º53'56 W |
| BG0523 | Bahía Guaymas | March 2005 | 27º55'06 N | 110º53'56 W |
| BG0524 | Bahía Guaymas | March 2005 | 27º55'06 N | 110º53'56 W |
| BG0525 | Bahía Guaymas | March 2005 | 27º55'06 N | 110º53'56 W |
| BG0526 | Bahía Guaymas | March 2005 | 27º55'06 N | 110º53'56 W |
| BG0527 | Bahía Guaymas | March 2005 | 27º55'06 N | 110º53'56 W |
| BG0528 | Bahía Guaymas | March 2005 | 27º55'06 N | 110º53'56 W |
| BG0529 | Bahía Guaymas | March 2005 | 27º55'06 N | 110º53'56 W |
| BG0530 | Bahía Guaymas | March 2005 | 27º55'06 N | 110º53'56 W |
| BG0531 | Bahía Guaymas | March 2005 | 27º55'06 N | 110º53'56 W |
| BG0532 | Bahía Guaymas | March 2005 | 27º55'06 N | 110º53'56 W |
| BG0533 | Bahía Guaymas | March 2005 | 27º55'06 N | 110º53'56 W |
| BG0534 | Bahía Guaymas | March 2005 | 27º55'06 N | 110º53'56 W |
| BG0535 | Bahía Guaymas | March 2005 | 27º55'06 N | 110º53'56 W |
| BG0536 | Bahía Guaymas | March 2005 | 27º55'06 N | 110º53'56 W |
| BG0537 | Bahía Guaymas | March 2005 | 27º55'06 N | 110º53'56 W |
| BG0538 | Bahía Guaymas | March 2005 | 27º55'06 N | 110º53'56 W |
| BG0539 | Bahía Guaymas | March 2005 | 27º55'06 N | 110º53'56 W |
| BG0540 | Bahía Guaymas | March 2005 | 27º55'06 N | 110º53'56 W |
| BG0541 | Bahía Guaymas | March 2005 | 27º55'06 N | 110º53'56 W |
| BG0542 | Bahía Guaymas | March 2005 | 27º55'06 N | 110º53'56 W |
| BG0543 | Bahía Guaymas | March 2005 | 27º55'06 N | 110º53'56 W |
| BG0544 | Bahía Guaymas | March 2005 | 27º55'06 N | 110º53'56 W |
| BG0545 | Bahía Guaymas | March 2005 | 27º55'06 N | 110º53'56 W |
| BG0546 | Bahía Guaymas | March 2005 | 27º55'06 N | 110º53'56 W |
| BG0547 | Bahía Guaymas | March 2005 | 27º55'06 N | 110º53'56 W |
| BG0548 | Bahía Guaymas | March 2005 | 27º55'06 N | 110º53'56 W |
| BG0549 | Bahía Guaymas | March 2005 | 27º55'06 N | 110º53'56 W |
| BG0550 | Bahía Guaymas | March 2005 | 27º55'06 N | 110º53'56 W |
| BG0551 | Bahía Guaymas | March 2005 | 27º55'06 N | 110º53'56 W |
| BG0552 | Bahía Guaymas | March 2005 | 27º55'06 N | 110º53'56 W |
| BG0553 | Bahía Guaymas | March 2005 | 27º55'06 N | 110º53'56 W |
| BG0554 | Bahía Guaymas | March 2005 | 27º55'06 N | 110º53'56 W |
| BG0555 | Bahía Guaymas | March 2005 | 27º55'06 N | 110º53'56 W |
| BG0556 | Bahía Guaymas | March 2005 | 27º55'06 N | 110º53'56 W |
| BG0557 | Bahía Guaymas | March 2005 | 27º55'06 N | 110º53'56 W |
| BG0558 | Bahía Guaymas | March 2005 | 27º55'06 N | 110º53'56 W |
| BG0559 | Bahía Guaymas | March 2005 | 27º55'06 N | 110º53'56 W |
| BG0560 | Bahía Guaymas | March 2005 | 27º55'06 N | 110º53'56 W |
| BG0561 | Bahía Guaymas | March 2005 | 27º55'06 N | 110º53'56 W |
| BG0562 | Bahía Guaymas | March 2005 | 27º55'06 N | 110º53'56 W |
| BG0563 | Bahía Guaymas | March 2005 | 27º55'06 N | 110º53'56 W |
| BG0564 | Bahía Guaymas | March 2005 | 27º55'06 N | 110º53'56 W |
| BG0565 | Bahía Guaymas | March 2005 | 27º55'06 N | 110º53'56 W |
| BG0566 | Bahía Guaymas | March 2005 | 27º55'06 N | 110º53'56 W |
| BG0567 | Bahía Guaymas | March 2005 | 27º55'06 N | 110º53'56 W |
| BG0568 | Bahía Guaymas | March 2005 | 27º55'06 N | 110º53'56 W |
| BG0569 | Bahía Guaymas | March 2005 | 27º55'06 N | 110º53'56 W |
| BG0570 | Bahía Guaymas | March 2005 | 27º55'06 N | 110º53'56 W |
| BG0601 | Bahía Guaymas | April 2006 | 27º55'06 N | 110º53'56 W |
| BG0602 | Bahía Guaymas | April 2006 | 27º55'06 N | 110º53'56 W |
| BG0603 | Bahía Guaymas | April 2006 | 27º55'06 N | 110º53'56 W |
| BG0604 | Bahía Guaymas | April 2006 | 27º55'06 N | 110º53'56 W |
| BG0605 | Bahía Guaymas | April 2006 | 27º55'06 N | 110º53'56 W |
| BG0606 | Bahía Guaymas | April 2006 | 27º55'06 N | 110º53'56 W |
| BG0607 | Bahía Guaymas | April 2006 | 27º55'06 N | 110º53'56 W |
| BG0608 | Bahía Guaymas | April 2006 | 27º55'06 N | 110º53'56 W |
| BG0609 | Bahía Guaymas | April 2006 | 27º55'06 N | 110º53'56 W |
| BG0610 | Bahía Guaymas | April 2006 | 27º55'06 N | 110º53'56 W |
| BG0611 | Bahía Guaymas | April 2006 | 27º55'06 N | 110º53'56 W |
| BG0612 | Bahía Guaymas | April 2006 | 27º55'06 N | 110º53'56 W |
| BG0613 | Bahía Guaymas | April 2006 | 27º55'06 N | 110º53'56 W |
| BG0614 | Bahía Guaymas | April 2006 | 27º55'06 N | 110º53'56 W |
| BG0615 | Bahía Guaymas | April 2006 | 27º55'06 N | 110º53'56 W |
| BG0616 | Bahía Guaymas | April 2006 | 27º55'06 N | 110º53'56 W |
| BG0617 | Bahía Guaymas | April 2006 | 27º55'06 N | 110º53'56 W |
| BG0618 | Bahía Guaymas | April 2006 | 27º55'06 N | 110º53'56 W |
| BG0619 | Bahía Guaymas | April 2006 | 27º55'06 N | 110º53'56 W |
| BG0620 | Bahía Guaymas | April 2006 | 27º55'06 N | 110º53'56 W |
| BG0621 | Bahía Guaymas | April 2006 | 27º55'06 N | 110º53'56 W |
| BG0622 | Bahía Guaymas | April 2006 | 27º55'06 N | 110º53'56 W |
| BG0623 | Bahía Guaymas | April 2006 | 27º55'06 N | 110º53'56 W |
| BG0624 | Bahía Guaymas | April 2006 | 27º55'06 N | 110º53'56 W |
| BG0625 | Bahía Guaymas | April 2006 | 27º55'06 N | 110º53'56 W |
| BG0626 | Bahía Guaymas | April 2006 | 27º55'06 N | 110º53'56 W |
| BG0627 | Bahía Guaymas | April 2006 | 27º55'06 N | 110º53'56 W |
| BG0628 | Bahía Guaymas | April 2006 | 27º55'06 N | 110º53'56 W |
| BG0629 | Bahía Guaymas | April 2006 | 27º55'06 N | 110º53'56 W |
| BG0630 | Bahía Guaymas | April 2006 | 27º55'06 N | 110º53'56 W |
| BG0631 | Bahía Guaymas | April 2006 | 27º55'06 N | 110º53'56 W |
| BG0632 | Bahía Guaymas | April 2006 | 27º55'06 N | 110º53'56 W |
| BG0633, | Bahía Guaymas | April 2006 | 27º55'06 N | 110º53'56 W |
| BG0634 | Bahía Guaymas | April 2006 | 27º55'06 N | 110º53'56 W |
| BG0635 | Bahía Guaymas | April 2006 | 27º55'06 N | 110º53'56 W |
| BG0636 | Bahía Guaymas | April 2006 | 27º55'06 N | 110º53'56 W |
| BG0637 | Bahía Guaymas | April 2006 | 27º55'06 N | 110º53'56 W |
| BG0638 | Bahía Guaymas | April 2006 | 27º55'06 N | 110º53'56 W |
| BG0639 | Bahía Guaymas | April 2006 | 27º55'06 N | 110º53'56 W |
| HT0501 | Huatabampo | April 2005 | 26º49'39 N | 119º38'32 W |
| HT0502 | Huatabampo | April 2005 | 26º49'39 N | 119º38'32 W |
| HT0503 | Huatabampo | April 2005 | 26º49'39 N | 119º38'32 W |
| HT0504 | Huatabampo | April 2005 | 26º49'39 N | 119º38'32 W |
| HT0505 | Huatabampo | April 2005 | 26º49'39 N | 119º38'32 W |
| HT0506 | Huatabampo | April 2005 | 26º49'39 N | 119º38'32 W |
| HT0507 | Huatabampo | April 2005 | 26º49'39 N | 119º38'32 W |
| HT0508 | Huatabampo | April 2005 | 26º49'39 N | 119º38'32 W |
| HT0509 | Huatabampo | April 2005 | 26º49'39 N | 119º38'32 W |
| HT0510 | Huatabampo | April 2005 | 26º49'39 N | 119º38'32 W |
| HT0511 | Huatabampo | April 2005 | 26º49'39 N | 119º38'32 W |
| HT0512 | Huatabampo | April 2005 | 26º49'39 N | 119º38'32 W |
| HT0513 | Huatabampo | April 2005 | 26º49'39 N | 119º38'32 W |
| HT0514 | Huatabampo | April 2005 | 26º49'39 N | 119º38'32 W |
| HT0515 | Huatabampo | April 2005 | 26º49'39 N | 119º38'32 W |
| HT0516 | Huatabampo | April 2005 | 26º49'39 N | 119º38'32 W |
| HT0517 | Huatabampo | April 2005 | 26º49'39 N | 119º38'32 W |
| HT0518 | Huatabampo | April 2005 | 26º49'39 N | 119º38'32 W |
| HT0519 | Huatabampo | April 2005 | 26º49'39 N | 119º38'32 W |
| HT0520 | Huatabampo | April 2005 | 26º49'39 N | 119º38'32 W |
| HT0521 | Huatabampo | April 2005 | 26º49'39 N | 119º38'32 W |
| HT0522 | Huatabampo | April 2005 | 26º49'39 N | 119º38'32 W |
| HT0523 | Huatabampo | April 2005 | 26º49'39 N | 119º38'32 W |
| HT0524 | Huatabampo | April 2005 | 26º49'39 N | 119º38'32 W |
| HT0525 | Huatabampo | April 2005 | 26º49'39 N | 119º38'32 W |
| HT0526 | Huatabampo | April 2005 | 26º49'39 N | 119º38'32 W |
| HT0527 | Huatabampo | April 2005 | 26º49'39 N | 119º38'32 W |
| HT0528 | Huatabampo | April 2005 | 26º49'39 N | 119º38'32 W |
