# Supplementary material for: Spatial and temporal genetic homogeneity of the Monterey Spanish mackerel, Scomberomorus concolor, in the Gulf of California
Source: PeerJ. 2016 Oct 25;4:e2583. doi: 10.7717/peerj.2583 (PMC5088583; doi:10.7717/peerj.2583)
Supplement: Table S2 [file peerj-04-2583-s003.docx]

| **Primer set** | **M13 Fluorescent dye** | **Loci** | **Primer sequence (5'–3')** | **Repeat motif** | **Size range (bp)** |
| --- | --- | --- | --- | --- | --- |
| 1 | FAM | *Sbr9* | F: GTCTTAAACGGAAGTGGGTT  R: GGAGCAGCGTACTACTTTGT | (GATA)_10_ | 311 – 367 |
|  |  | *Sbr24* | F: ATTTTGATGTTTCGCCATGAA  R: GCCCACCAACAACTGCTATTA | (CAGA)_8_ | 180 – 216 |
|  |  | *Sbr35* | F: TGCTCCCTCTGCTTCTGTAAC  R: ATGGTACACACATGCACCTCA | (CA)_14_ | 118 – 174 |
| 2 | VIC | *Sca44* | F: ATGGCCAAATGGCACATAATCA  R: GGGCAGCTCCATGGGTCTGAGT | (GACA)_9_ | 160 – 196 |
|  |  | *Sbr28* | F: TGGCTGATTTTGTTGATGTTG  R: TGCAGATTCTACAGGCGAGAT | (GATA)_6_ | 327 – 363 |
| 3 | NED | *Sbr18* | F: TCCTGCTGCAGTATGTGAATG  R: TTTGTCCCTCTCCCTAATGCT | (GACA)_6_(GATA)_12_ | 276 – 452 |
|  |  | *Sbr36* | F: CCTGCTAGGCAGTGCAATATC  R: TCACCCTAAGAGAGGGAGAGG | (GATA)_5_4bp(GATA)_2_ | 152 – 176 |
| 4 | PET | *Sbr26* | F: TACCTGAAAGGCATCCAACAC  R: ATTGCAGCACTGATGTGTGTC | (CA)_15_ | 253 – 297 |
|  |  | *Sni26* | F: AACAACCTCTTAGGAGCTGACG  R: GATCGCTCTCAGATACATGCC | (CA)_15_ | 126 – 184 |

**Summary data for the microsatellites characterized for *S. concolor* individuals.**

Multiplex–PCR sets. *Sbr*, *Sca* and *Sni* loci were previously isolated in *Scomberomorus brasiliensis* (*Renshaw et al., 2009*), *S. cavalla* (*Broughton, Stewart & Gold, 2002*) and *S. niphonius* *(Yokoyama et al., 2006)*, respectively, and characterized in *S. concolor* in the present study. Forward primer sequence (F), reverse primer sequence (R). All the multiplex reactions were performed at annealing temperature of 56ºC.
