# Supplementary material for: Spatial and temporal genetic homogeneity of the Monterey Spanish mackerel, Scomberomorus concolor, in the Gulf of California
Source: PeerJ. 2016 Oct 25;4:e2583. doi: 10.7717/peerj.2583 (PMC5088583; doi:10.7717/peerj.2583)
Supplement: Table S3 — Summary statistics for nine microsatellite loci amplified for S. concolor for each location and for the entire population in the GC. [file peerj-04-2583-s004.docx]

Summary statistics for nine microsatellite loci amplified for *S. concolor* for each location and for the entire population in the GC.

| **Location** | **Locus** | | | | | | | | | **Average** |
| --- | --- | --- | --- | --- | --- | --- | --- | --- | --- | --- |
|  | ***Sca44*** | ***Sbr9*** | ***Sbr18*** | ***Sbr24*** | ***Sbr26*** | ***Sbr28*** | ***Sbr35*** | ***Sbr36*** | ***Sn26*** | **across loci** |
| **SF** | | | | | | | | | | |
| *n* | 60 | 64 | 61 | 67 | 65 | 66 | 68 | 68 | 56 |  |
| *A* | 6 | 12 | 33 | 8 | 14 | 6 | 17 | 6 | 21 | 13.67 |
| *Ar* | 5.84 | 9.88 | 23.70 | 6.59 | 9.78 | 4.30 | 10.30 | 5.54 | 17.53 | 10.38 |
| *H_O_* | 0.567 | 0.844 | 0.934 | 0.552 | 0.769 | 0.303 | 0.809 | 0.676 | 0.964 | 0.713 |
| *H_E_* | 0.632 | 0.861 | 0.96 | 0.619 | 0.748 | 0.31 | 0.799 | 0.717 | 0.936 | 0.731 |
| *HW* | 0.098 | 0.993 | 0.247 | 0.122 | 0.497 | 0.371 | 0.212 | 0.441 | 0.750 |  |
| *F_IS_* | 0.104 | 0.020 | 0.027 | 0.109 | -0.028 | 0.021 | -0.013 | 0.057 | -0.030 |  |
| **SC** | | | | | | | | | | |
| *n* | 26 | 29 | 29 | 25 | 29 | 29 | 29 | 29 | 24 |  |
| *A* | 7 | 11 | 28 | 6 | 11 | 4 | 12 | 6 | 18 | 11.44 |
| *Ar* | 6.91 | 10.59 | 25.70 | 5.92 | 10.71 | 3.80 | 10.74 | 5.95 | 18.00 | 10.92 |
| *H_O_* | 0.654 | 0.931 | 0.862 | 0.480 | 0.655 | 0.310 | 0.724 | 0.586 | 0.875 | 0.675 |
| *H_E_* | 0.637 | 0.874 | 0.970 | 0.534 | 0.717 | 0.279 | 0.716 | 0.722 | 0.935 | 0.709 |
| *HW* | 0.692 | 0.628 | **0.004** | 0.130 | 0.117 | 1.000 | 0.463 | 0.404 | 0.253 |  |
| *F_IS_* | -0.027 | -0.066 | 0.113 | 0.103 | 0.088 | -0.115 | -0.011 | 0.190 | 0.066 |  |
| **PP** | | | | | | | | | | |
| *n* | 64 | 71 | 68 | 76 | 72 | 75 | 76 | 68 | 62 |  |
| *A* | 7 | 13 | 31 | 9 | 15 | 4 | 13 | 7 | 24 | 13.67 |
| *Ar* | 6.35 | 11.05 | 23.70 | 6.10 | 10.61 | 3.30 | 9.33 | 5.61 | 19.50 | 10.62 |
| *H_O_* | 0.625 | 0.901 | 0.868 | 0.579 | 0.792 | 0.333 | 0.816 | 0.721 | 0.887 | 0.725 |
| *H_E_* | 0.623 | 0.884 | 0.963 | 0.633 | 0.787 | 0.314 | 0.788 | 0.709 | 0.95 | 0.739 |
| *HW* | 0.455 | 0.714 | 0.021 | 0.532 | 0.169 | 0.395 | 0.122 | 0.334 | 0.010 |  |
| *F_IS_* | -0.004 | -0.020 | **0.100** | 0.087 | -0.006 | -0.063 | -0.036 | -0.017 | 0.067 |  |
| **PL** | | | | | | | | | | |
| *n* | 28 | 30 | 30 | 30 | 30 | 30 | 30 | 30 | 30 |  |
| *A* | 5 | 9 | 30 | 6 | 12 | 5 | 12 | 6 | 16 | 11.22 |
| *Ar* | 4.86 | 8.96 | 26.71 | 5.56 | 11.08 | 4.60 | 10.40 | 5.60 | 14.88 | 10.29 |
| *H_O_* | 0.607 | 0.733 | 1.000 | 0.733 | 0.833 | 0.400 | 0.767 | 0.633 | 0.900 | 0.734 |
| *H_E_* | 0.694 | 0.882 | 0.968 | 0.632 | 0.779 | 0.419 | 0.790 | 0.690 | 0.911 | 0.751 |
| *HW* | 0.422 | 0.138 | 0.880 | 0.831 | 0.235 | 0.705 | 0.546 | 0.369 | 0.216 |  |
| *F_IS_* | 0.127 | 0.171 | -0.033 | -0.164 | -0.072 | 0.047 | 0.030 | 0.083 | 0.012 |  |
| **BK** | | | | | | | | | | |
| *n* | 133 | 127 | 120 | 138 | 134 | 135 | 140 | 132 | 120 |  |
| *A* | 9 | 14 | 38 | 9 | 20 | 6 | 16 | 6 | 25 | 15.89 |
| *Ar* | 6.81 | 10.08 | 23.95 | 5.72 | 12.88 | 3.87 | 8.01 | 5.37 | 18.21 | 10.54 |
| *H_O_* | 0.579 | 0.795 | 0.917 | 0.609 | 0.813 | 0.281 | 0.721 | 0.742 | 0.950 | 0.712 |
| *H_E_* | 0.663 | 0.866 | 0.961 | 0.607 | 0.828 | 0.306 | 0.729 | 0.721 | 0.936 | 0.735 |
| *HW* | 0.046 | 0.221 | 0.143 | 0.977 | 0.795 | 0.079 | 0.078 | 0.523 | 0.016 |  |
| *F_IS_* | 0.127 | 0.082 | 0.046 | -0.004 | 0.018 | 0.080 | 0.011 | -0.030 | -0.015 |  |
| **BG** | | | | | | | | | | |
| *n* | 96 | 104 | 106 | 106 | 106 | 105 | 107 | 107 | 97 |  |
| *A* | 8 | 11 | 38 | 8 | 17 | 7 | 18 | 6 | 25 | 15.33 |
| *Ar* | 7.02 | 9.28 | 24.28 | 5.84 | 12.50 | 4.07 | 9.97 | 5.36 | 18.23 | 10.73 |
| *H_O_* | 0.656 | 0.827 | 0.877 | 0.679 | 0.830 | 0.343 | 0.822 | 0.710 | 0.938 | 0.742 |
| *H_E_* | 0.725 | 0.873 | 0.962 | 0.672 | 0.844 | 0.378 | 0.788 | 0.717 | 0.937 | 0.766 |
| *HW* | 0.013 | 0.881 | 0.016 | 0.025 | 0.314 | 0.131 | 0.818 | 0.925 | 0.834 |  |
| *F_IS_* | 0.095 | 0.053 | **0.089** | -0.011 | 0.016 | 0.092 | -0.044 | 0.009 | -0.001 |  |
| **HT** | | | | | | | | | | |
| *n* | 27 | 26 | 28 | 28 | 26 | 28 | 28 | 28 | 27 |  |
| *A* | 7 | 10 | 26 | 6 | 8 | 3 | 9 | 5 | 19 | 10.33 |
| *Ar* | 6.8918 | 9.91 | 24.29 | 5.71 | 7.76 | 3.00 | 8.55 | 4.86 | 18.07 | 9.89 |
| *H_O_* | 0.741 | 0.808 | 0.929 | 0.679 | 0.615 | 0.286 | 0.857 | 0.786 | 1.000 | 0.744 |
| *H_E_* | 0.726 | 0.879 | 0.962 | 0.645 | 0.593 | 0.260 | 0.782 | 0.707 | 0.930 | 0.720 |
| *HW* | 0.918 | 0.416 | 0.640 | 0.547 | 0.656 | 1.000 | 0.799 | 0.350 | 0.921 |  |
| *F_IS_* | –0.021 | 0.082 | 0.035 | -0.052 | -0.039 | -0.102 | -0.098 | -0.113 | -0.077 |  |
| **GC** | | | | | | | | | | |
| *n* | 434 | 451 | 442 | 470 | 462 | 468 | 478 | 462 | 416 |  |
| *A* | 10 | 15 | 43 | 10 | 20 | 9 | 26 | 7 | 28 |  |
| *Ar* | 9.92 | 14.85 | 42.77 | 9.88 | 19.89 | 8.65 | 25.29 | 6.90 | 28.00 |  |
| *H_O_* | 0.618 | 0.831 | 0.905 | 0.617 | 0.788 | 0.316 | 0.782 | 0.708 | 0.935 |  |
| *H_E_* | 0.673 | 0.874 | 0.963 | 0.627 | 0.796 | 0.326 | 0.770 | 0.713 | 0.940 |  |
| *HW* | 0.049 | 0.679 | 0.043 | 0.705 | 0.217 | 0.063 | 0.077 | 0.923 | **0.002** |  |
| *F_IS_* | **0.083** | 0.048 | **0.060** | 0.017 | 0.011 | 0.029 | -0.017 | 0.008 | 0.006 |  |

Number of individuals (*n*), number of alleles (*A*), allelic richness per locus and sample based on a minimum sample size of 24 individuals (*Ar*), observed heterozygosity (*H_O_*), expected heterozygosity (*H_E_*), Hardy-Weinberg probability values (*HW)* and fixation index *(F_IS_).* HW probability values in bold represent significant deviations from equilibrium and *F_IS_* values in bold correspond to heterozygosity deficit after Bonferroni correction (α = 0.05/9 = 0.0056).
