# Supplementary material for: Spatial and temporal genetic homogeneity of the Monterey Spanish mackerel, Scomberomorus concolor, in the Gulf of California
Source: PeerJ. 2016 Oct 25;4:e2583. doi: 10.7717/peerj.2583 (PMC5088583; doi:10.7717/peerj.2583)
Supplement: Table S4 — Results of FST pairwise sample comparisons over temporal collections. (A) San Felipe (SF) collections. (B) Puerto Peñasco (PP) comparisons. (C) Bahía Kino (BK) comparisons and (D) Bahía Guaymas (BG) comparisons. None FST value showed significant differences (p < 0.05). [file peerj-04-2583-s005.docx]

Results of F_ST_ pairwise sample comparisons over temporal collections. **A.** San Felipe (SF) collections. **B.** Puerto Peñasco (PP) comparisons. **C.** Bahía Kino (BK) comparisons and **D.** Bahía Guaymas (BG) comparisons. No F_ST_ values showed significant differences (p < 0.05).

**A.**

|  | **SF06** | **SF08** |
| --- | --- | --- |
| **SF06** | * |  |
| **SF08** | 0.0 | * |

|  | **PP06** | **PP07** | **PP08** |
| --- | --- | --- | --- |
| **PP06** | * |  |  |
| **PP07** | 0.0 | * |  |
| **PP08** | 0.006 | 0.009 | * |

**B.**

|  | **BK05** | **BK06** | **BK07** |
| --- | --- | --- | --- |
| **BK05** | * |  |  |
| **BK06** | 0.003 | * |  |
| **BK07** | 0.0 | 0.0 | * |

**C.**

|  | **BG05** | **BG06** |
| --- | --- | --- |
| **BG05** | * |  |
| **BG06** | 0.003 | * |

**D.**
